# Can Current Metrics Characterise or Differentiate Between Elite Track and Field Athletes Within the Sprints, Hurdles, High Jump, Long Jump and Triple Jump? A Systematic Review

**DOI:** 10.1007/s40279-025-02284-5

**Published:** 2025-07-28

**Authors:** Kirstie J. Turner, Danielle Trowell, Emma L. Millett, Jason Bonacci, Tarryn M. Elvish, Peter Kremer, Craig Pickering, Natalie Saunders

**Affiliations:** 1 Centre for Sport Research, Deakin University, 75 Pigdons Road, Waurn Ponds, VIC 3216 Australia; 2 Australian Athletics, Melbourne, VIC Australia; 3 NSW Institute of Sport, Sydney, NSW Australia

## Abstract

**Background:**

High-performance sport programs aim to effectively identify talented athletes with the greatest potential for achieving podium success at benchmark events such as the World Championships or Olympic Games. However, not every talented athlete will go on to achieve success at the highest level, and the characteristics used to identify athletes are not well understood.

**Objective:**

The aim of this study was to systematically review the literature to understand parameters that may be associated with podium success at benchmark events in speed/power-based track and field events.

**Methods:**

A systematic search of articles was performed across Scopus, PubMed, EBSCOhost (Academic Search Complete and SPORTDiscus) and Web of Science based on Preferred Reporting Items for Systematic Reviews and Meta-analyses (PRISMA) guidelines. Studies were eligible if they: (a) were published in English; (b) met the minimum performance criteria; (c) included track and field athletes in the 100–400 m sprint, 100/110 m hurdles, high jump, long jump or triple jump; and (d) examined biological or physical performance characteristics or determinants. Risk of bias was assessed using a 16-item checklist. Mean and standard deviations for sample characteristics and performance outcomes were analysed where possible. Articles based on competition data allowed for a comparison between medallists and non-medallists for each event group.

**Results:**

Forty-four articles were included in the systematic review. Of these 44 articles, 36 were competition analyses, five were field analyses (i.e. training) and three were laboratory analyses. Across the eight event groups investigated, ~ 509 athletes were evaluated with 323 athletes having individual competition data available for analyses. The key findings of reviewed studies were organised by event group. Medallists in the 100 m for both men and women achieved a greater peak velocity (men 0.46 m/s; women 0.22 m/s) and were better able to limit the deceleration phase. Compared with non-medallists in the 200 m, medallists were faster across the first 100 m for women (0.25 s) and second 50 m (0.10 s) and final 50 m for men (0.11 s). Medallists in the 400 m were faster in the final 150 m for both men (0.69 s) and women (0.58 s), compared with non-medallists. For both men and women in the sprint hurdles, the greatest differences between medallists and non-medallists emerged in the hurdle 9–10 unit time and run to the finish line time. Medallists in the men’s 400-m hurdles maintained a 13-step count between hurdles for one hurdle longer than non-medallists (who shifted to 14 steps), while finalists in the women’s event all followed a similar step pattern. The greatest differences in hurdle unit time between medallists and non-medallists emerged from hurdle five for women (medallists 0.18–0.22 s faster) and hurdle six for men (medallists 0.06–0.13 s faster). In the high jump, medallists for both men and women recorded a greater vertical velocity at take-off. Medallists also achieved a peak centre of mass height ~ 10 cm higher than their best bar clearance, while non medallists achieved a height ~ 5 cm higher. Medallists in the long jump for both men and women achieved a greater horizontal velocity at touchdown prior to take-off (men 0.41 m/s; women 0.30 m/s) than non-medallists. Male medallists also achieved a greater horizontal velocity at take-off (0.29 m/s) while female medallists achieved a greater vertical velocity (0.23 m/s). Male triple jump medallists were jump dominant while non medallists were hop dominant and there was no difference for women. For both men and women, medallists maintained a greater horizontal velocity across the triple jump phases.

**Conclusions:**

Across the eight event groups examined, various characteristics that may be associated with benchmark event podium success were identified. However, their ability to reliably differentiate between medal winners and non-medal winners remains inconclusive. While these characteristics may not be able to definitively identify podium success, they can be used to inform performance testing or areas for development.

## Key Points

**Table:** 

Many characteristics have been assessed in the literature; however, exactly which characteristics can be used to differentiate between medallists and non-medallists remains unclear.
The most notable differences in the 100 m emerged in the latter stages of the race, suggesting medallists are better able to maintain their velocity and limit their deceleration phase.
Medallists in the women’s 200 m held a faster pace across the initial stages of the race, but not the final 50 m, indicating the first 150 m may be more important in deciding a race outcome.
In the men’s 400 m, medallists were slower across the first 100 m but faster across the final 200 m compared with non-medallists, suggesting medallists may implement a better pacing strategy and can maintain a greater velocity under fatigue.

## Introduction

High-performance, or elite sport, refers to the highest level of competition with an emphasis on performing well at prestigious competitions such as the World Championships or Olympic Games. Many elite athletes begin their sporting career as talented individuals, possessing an innate ability to excel in a particular event that requires special skills and training [1]. Debate exists around defining ‘talent’ and is likely to have a nuanced meaning in different contexts [2]. For those working in high-performance sport or athlete development, talent has been suggested to be defined broadly as the potential to achieve success at a higher level of competition in the future (e.g. senior success) [3]. Talented individuals dedicate a significant amount of time to developing their skills to perform at the highest level on the global stage [4]. Many talented individuals, however, will never achieve medal success at the highest level or even gain selection for a senior national team [5]. Current performance is often used as a proxy for potential [2], with potential referring to an athlete’s capacity to develop their current attributes into those required for success at the highest level of competition. Specifically, within track and field, success at a junior level is a poor predictor of senior athletic potential. Less than 20% of junior sprinters or jumpers ranked in the global top 50 go on to be ranked within the global senior top 50 [6, 7]. So, while potential is often inferred from current performance, we need to consider the multifaceted nature of athletic development, which includes biological, psychological and social factors that may influence an athlete’s trajectory. Acknowledging the challenge of defining potential, and the low conversion rate from junior to senior success, it is crucial to explore methods that go beyond simple performance markers to support athlete development. Therefore, it is in the interests of high-performance sport programs to be able to effectively identify athletes with the greatest potential for achieving podium success at benchmark events (BME) such as the World Championships or Olympic Games.

Sports that comprise multiple discrete disciplines will often group such disciplines as being of an endurance nature or of a speed/power nature separately owing to the large variation in demands. In athletics, track and field is a broad term that encompasses a multitude of disciplines. These disciplines include running (100–10,000 m, 100/110-m and 400-m hurdles and 3000-m steeplechase), jumping (long, triple, high and pole vault), throwing (discus, javelin, shot put and hammer), racewalking and the multi-events (decathlon and heptathlon). This review focuses on events within the running and jumping disciplines; specifically, those within the speed/power domain (100–400 m sprint, 100–400 m hurdles, high jump, long jump and triple jump) specific to able-bodied athletics.

Researchers have proposed several factors that can contribute to more successful performances across speed/power-based events. However, characteristics that may differentiate athletes or characterise those who achieve podium success are not well understood. Factors that can contribute to faster sprint performance include maximal adenosine triphosphate turnover rate, glycolytic metabolism [8], maximal running velocity [9], leg stiffness [10], muscle composition, stride length and stride frequency [8]. Specifically, stride length was reported to have a greater association with sprint performance in elite men, while stride frequency was reported to have a greater impact on performance in elite women [9]. It is also important to note that the term ‘elite’ is inconsistently applied in research and often can encompass athletes from a youth level to Olympic medallists [11]. For example, a review by Swann et al. [12] identified eight approaches to define the ‘elite athlete’, which ranged from Olympic champions to regional competitors with elements considered in the classification of athletes including experience, success, training and involvement in talent development programmes. More recently, a six-tiered Participation Classification Framework has been proposed that guides the classification of athletes from sedentary to world class [13]. This framework considers factors such as world ranking, performance relative to respective world record or world leading performance, highest level of competition, skill level and training status [13].

In addition to the factors influencing elite sprint performance, it is important to consider elements that may contribute to the hurdles, long jump, triple jump and high jump performance. For instance, in national-level performers, horizontal velocity, vertical velocity and take-off angle have all been noted as important contributors to hurdle performance [14]. Hurdle stride length, in particular a longer hurdle stride length, is desirable as it allows an athlete to maintain a higher horizontal velocity [15]. Long and triple jump performance can be influenced by a faster approach and take-off velocity [16], as well as the maintenance of velocity through the hop, step and jump phases of the triple jump [17, 18]. In the high jump, approach velocity and vertical take-off velocity are two contributors to successful performance [19, 20]. For field-based events such as the high or triple jump, a greater standing height is often required just to be competitive but unlikely to be the difference between those who win and those who do not at the elite level [21]. The assessment of characteristics offers valuable insights for athletic potential but has limitations, particularly in predicting long-term success. While it provides objective data, it cannot fully capture the complexity of athletic potential, highlighting the need for evidence-informed decisions despite the challenges in available data. Additionally, despite numerous studies examining the relationship between various characteristics and performance, a gap remains in the literature with regard to the association of these characteristics to success at BME. The literature that has attempted to characterise the elite population often focuses on comparing higher performing athletes relative to their sub-elite or national-level counterparts [22, 23]. It therefore remains unclear whether observed differences between performers can be attributed to varying levels of athletic ability or if these characteristics are truly capable of differentiating between athletes capable of achieving BME podium success.

To date, no systematic review has investigated athletic characteristics that may be associated with BME podium success in able-bodied athletes within speed/power-based track and field events. The identification of these characteristics may allow for more targeted development in emerging athletes and a more focused approach to performance testing related to talent development. Therefore, this review aimed to systematically identify characteristics relative to the event group that: (a) are associated with benchmark event podium success; and/or (b) differentiate medallists and non-medallists at BME.

## Methods

### Search Strategy

The search strategy was developed in collaboration with members of the research team, which included two PhD-qualified industry experts to ensure search terms aligned with the research question and were broad enough to capture relevant articles. Potential articles were identified through a systematic search of four electronic databases that included all publication years (i.e. inception to August 2022). Scopus, PubMed, EBSCOhost (Academic Search Complete and SPORTDiscus) and Web of Science, most commonly used for sport-related research, were the databases used to conduct a systematic literature search. The operators “AND”, “OR” and “NOT” were applied to terms such as “elite OR world class OR Olympic” AND “track and field” AND “sprinting OR hurdling OR long jump OR triple jump OR high jump” AND “performance OR characteristics OR determinants”. Reference lists from studies involving track-and-field events in the speed/power domain were also manually searched.

For the screening process, the eligibility criteria were first applied to the title and abstract only. Following this, the selection criteria were then applied to the full text of the remaining articles. Articles were only included in the review if they met all the inclusion criteria following the full-text screening. Non-relevant titles and abstracts were excluded. The screening process was undertaken by a member of the research team (KT) to identify potentially relevant papers, with queries being clarified and checked by the senior researcher (NS).

### Eligibility Criteria and Inclusion/Exclusion Criteria

The Preferred Reporting Items for Systematic Review and Meta Analyses (PRISMA) guidelines were adopted to guide the screening of studies assessing the performance of track and field speed/power athletes [24]. Inclusion criteria were: (a) full-text articles in English language; (b) participants met minimum performance criteria to be classified as elite (Table 1); (c) participants included track-and-field athletes in speed/power events; and (d) study examined biological or physical performance characteristics or determinants. Considering the Participant Classification Framework [13], performance criteria (Table 1) were based on the minimum requirements for automatic qualification standard for the World Championships or Olympic Games across the 2016–20 Olympiad to ensure data captured were from the highest-performing athletes. The automatic qualification standards for the World Championships and Olympic Games are established by World Athletics with the aim of 50% of athletes competing at these events qualifying via the standard. While this does not account for a competitive context and may overemphasise physical performance, it provides an objective approach to capturing data relative to athletes at the highest level. In the absence of being able to compare between medallists and non-medallists, data were also considered for athletes who achieved the automatic qualification standards (Table 1) and therefore had the potential to achieve podium success. Exclusion criteria were: (a) study was based on training load monitoring or return from injury; (b) genetics or genetic testing; (c) supplementation, doping or dietary intervention; (d) training technology; and (e) sample contained Paralympic athletes. Speed/power athletes were defined as those competing in 100-m sprint, 200-m sprint, 400-m sprint, 100/110-m hurdles, 400-m hurdles, high jump, long jump and triple jump.

**Table 1:** Minimum performance criteria for inclusion in the systematic review

Event	Men	Women
100 m (s)	10.16	11.32
200 m (s)	20.50	23.20
400 m (s)	45.50	52.20
110/100-m hurdles (s)	13.48	13.00
400-m hurdles (s)	49.40	56.20
High jump (m)	2.29	1.93
Long jump (m)	8.15	6.70
Triple jump (m)	16.80	14.10

*m* metres, *s* seconds

### Quality Assessment

A 16-item checklist, previously utilised in systematic reviews conducted in soccer and canoe slalom [25–27], was selected as it better aligned with the present study objectives by allowing comparisons between competition-based and field- or laboratory-based research. Other common tools did not allow for the same comparison of athlete performance data collected across multiple environments. The selected checklist was adapted by Sarmento et al. [25] from a risk-of-bias quality form presented by Law et al. [28] for the specific context of research related to a performance analysis. This checklist includes items related to the: (1) study purpose; (2) literature background; (3) appropriate design; (4) sample details; (5) sample size justification; (6) informed consent; (7) reliability of outcomes; (8) validity of outcomes; (9) methods; (10) statistical significance of presented results; (11) appropriate analysis; (12) practical importance; (13) reporting of dropouts; (14) appropriate conclusions; (15) practical applications; and (16) study limitations. Questions were given a score of 0 (no) or 1 (yes), except for question 13 that also included an option for ‘not applicable’. Answers were summed and the final score for each study was divided by the maximum a study could achieve and expressed as a percentage. To make a fair comparison between studies of differing design, a percentage score was calculated to measure methodological quality [25]. Studies scoring ≤ 50% were classified as low quality, studies scoring 51–75% were classified as good quality and studies scoring > 75% were classified as excellent quality [25]. Eligible studies were assessed by three members of the research team (KT, TE and NS). Following the independent scoring of studies, discrepancies were reviewed and discussed by the three members of the research team to clarify the interpretation of criteria and reach a consensus.

### Data Extraction

The data extracted consisted of: (a) characteristics of the study (i.e. authors, year, experimental group); (b) participant characteristics (anthropometrics, personal best, season best or analysed performance result, sample size, sex); (c) track-and-field event (i.e. 100/200/400-m sprint, 100/110/400-m hurdles, high/long/triple jump); and (d) details of performance results and outcome measures (i.e. results from competition- and/or laboratory/field-based analyses). Competition data refer to those collected during official sporting events, which typically involve maximal effort performances that represent the athlete’s performance capability in an uncontrolled environment. Laboratory-based data refer to those collected in a controlled indoor environment using standardised protocols and equipment. Field-based data refer to those collected in an athlete’s training or performance setting, with more control over the environment than competition-based data collection but less than laboratory based. The data extraction process was completed by one member of the research team (KT) with the senior researcher (NS) supervising the process, to ensure accuracy, and providing clarity on any queries.

### Statistical Analysis

Mean and standard deviations for sample characteristics and performance results for individual athlete competition variables were analysed using Microsoft Excel (Version 2202; Microsoft Corporation, Redmond, WA, USA). Competition-based analysis presented in a report or a peer-reviewed literature format allows for individual athlete’s performance at BME events, including World Championship and Olympic finals, to be examined. Competition-based analyses afforded the ability to group and report data as medallists and non-medallists to better address the aims of this review. A meta-analysis was initially considered for this review and attempts were made; however, it was ultimately not feasible because of the nature of competition-based research, which often analyses repeat populations (i.e. athletes are analysed across multiple studies as a result of competing in two or more finals that were analysed). For athletes with multiple results, only their best performance (i.e. fastest time, longest jump or highest clearance) was included in the analysis. This approach was adopted to avoid a disproportionate representation of top athletes (e.g. Usain Bolt) in the literature to address the aims of this research. However, this yielded insufficient data, irrespective of sufficient research studies, to conduct the meta-analysis.

## Results

### Search and Quality Assessment

**Fig. 1:**
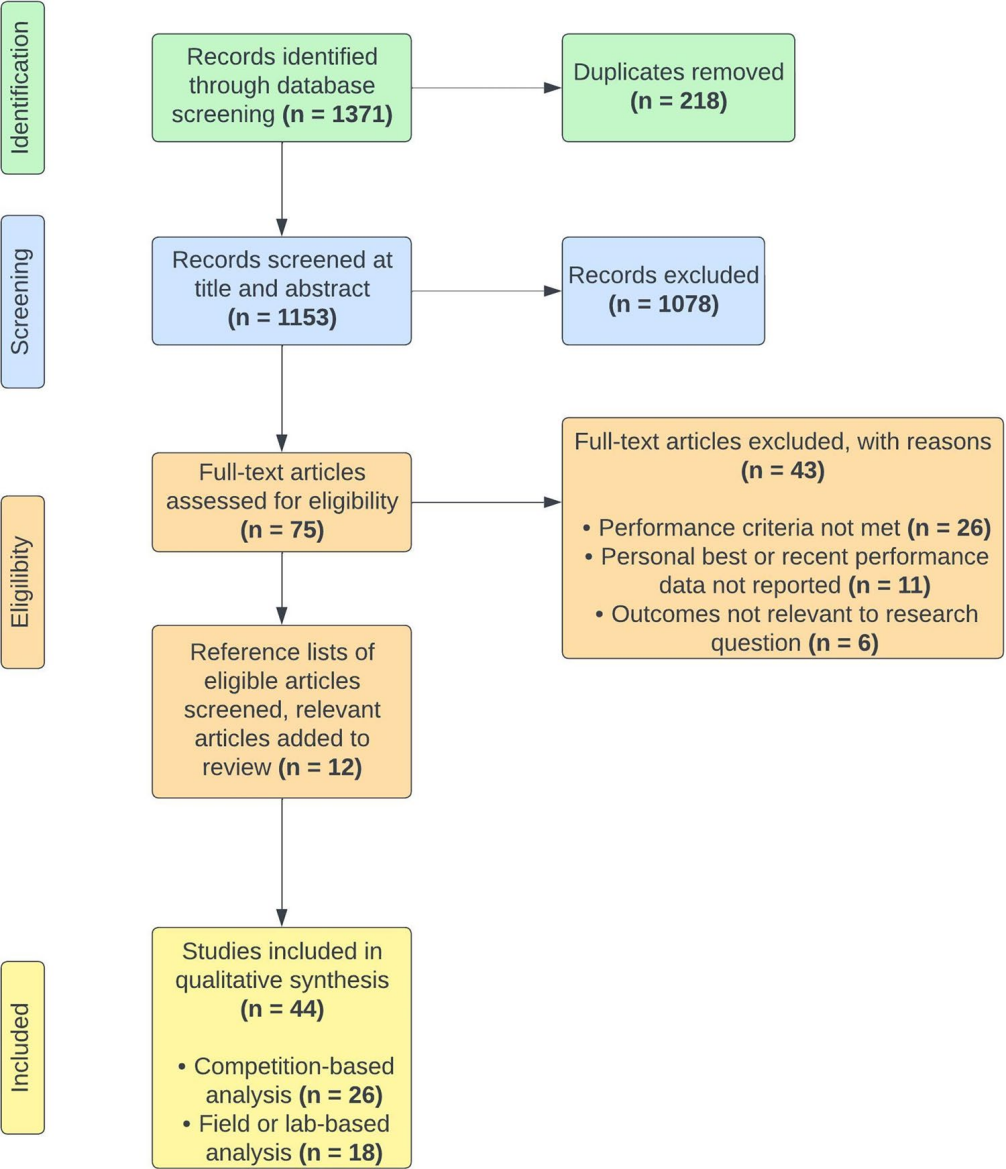
Study selection flow diagram

The initial database search identified a total of 1371 potentially relevant journal articles (Fig. 1). Following removal of 218 duplicates, titles and abstracts of 1153 articles were screened relative to the inclusion and exclusion criteria. This resulted in 75 eligible studies. The full-text versions for the remaining articles were screened and an additional 43 studies were removed. These studies were excluded for: (1) performance criteria were not met; (2) personal best of recent performance standard not reported; and (3) outcomes were not relevant to the research question. Reference lists from eligible articles were screened, resulting in a further 12 articles meeting the criteria for inclusion in the review.

The final number of studies included was 44 (Table 2). Overall, ~ 509 athletes (Paruzel-Dyja et al. [9] did not report the number of athletes) were evaluated with 323 of these athletes having competition data available for analyses (182 men, 141 women). The mean (± standard deviation) quality score for included studies was 66.5% (± 13.7%) and they were classified as having good methodological quality.

**Table 2:** Characteristics, measures and quality score of included studies across both competition and laboratory-based settings organised by event group

Study	Event	Environment	Performance level (SD)			Quality score (%)
			Men		Women	
Ae et al. [30]	100-m sprint	Competition	9.97 s (0.11), *n* = 8			60
Ciacci et al. [38]	100-m sprint	Competition	10.03 s (0.14), *n* = 4		11.1 s (0.17), *n* = 4	87
Čoh et al. [35]	100-m sprint	Competition	10.10 s (0.16), *n* = 7			67
Colyer et al. [40]	100-m sprint	Laboratory	< 10.15 s (N/A), *n* = 5			87
Krzysztof and Mero [37]	100-m sprint	Competition	Bolt: 9.69, 9.58, 9.63 s Other finalists 2008: 9.96 (0.05), *n* = 7 Other finalists 2009: 9.91 (0.10), *n* = 7 Other finalists 2012: 9.86 (0.10), *n* = 6			60
Miller et al. [23]	100-m sprint	Laboratory	9.99 s (0.07), *n* = 5			93
Moravec et al. [29]	100-m sprint	Competition	10.11 s (0.18), *n* = 7		11.07 s (0.09), *n* = 8	60
Paruzel-Dyja et al. [9]	100-m sprint	Competition	10.15 s (0.07), *n* = N/A		11.11 s (0.1), *n* = N/A	67
Salo et al. [36]	100-m sprint	Competition	10.09 s (0.05), *n* = 8			60
Slawinski et al. [39]	100-m sprint	Competition	9.96 s (0.16), *n* = 34		11.01 (0.16), *n* = 36	73
Taylor and Beneke [34]	100-m sprint	Competition	9.66 s (0.07), *n* = 3			87
Mann and Herman [41]	200-m sprint	Competition	20.20 s (0.57), *n* = 3			60
Ferro et al. [32]	100–400 m sprint	Competition	100 m: 10.00 s (0.14), *n* = 8 200 m: 20.20 s (0.21), *n* = 7 400 m: 44.45 s (0.61), *n* = 8		100 m: 10.93 s (0.15), *n* = 8 200 m: 22.30 s (0.26), *n* = 8 400 m: 50.24 s (0.44), *n* = 8	60
Amara et al. [47]	110-m hurdles	Competition	12.91 s, *n* = 1			80
Charalambous et al. [49]	110-m hurdles	Field	13.48, *n* = 1			80
Čoh and Dolonec [46]	100-m hurdles	Competition			12.75 s, *n* = 1	40
Čoh et al. [48]	110-m hurdles	Competition	12.89 s (0.03), *n* = 2			87
Hucklekemkes [43]	100-m hurdles	Competition			12.59 (0.30), *n* = 2	47
Mann and Herman [42]	100-m hurdles	Competition			13.04 s (0.31), *n* = 3	53
Rash et al. [50]	100-m hurdles	Field			12.85 s (0.04), *n* = 6	53
Tsiokanos et al. [44]	100-m hurdles	Competition			12.65 s (0.14), *n* = 14	87
Tsiokanos et al. [45]	110-m hurdles	Competition	13.29 s (0.22), *n* = 15			87
Ditrolio and Marini [51]	400-m hurdles	Competition	48.18 s (0.56), *n* = 8			53
Otsuka and Isaka [52]	400-m hurdles	Competition	World class: 47.71 s (0.44), *n* = 13 Elite: 49.28 s (0.41), *n* = 14			80
Ae et al. [55]	High jump	Competition	2.35 m (0.00), *n* = 3			67
Brüggemann and Loch [53]	High jump	Competition	2.34 m (0.03), *n* = 8		1.96 m (0.05), *n* = 8	47
Dapena et al. [57]	High jump	Competition	2.32 m (0.04), *n* = 40			53
Greig and Yeadon [58]	High jump	Field	2.32 m, *n* = 1			73
Isolehto et al. [54]	High jump	Competition	2.29 m (0.02), *n* = 10			73
Lees et al. [19]	High jump	Competition	2.37 m (0.04), *n* = 6			87
Pavlović 2017 [56]	High jump	Competition	2.32 m (0.00), *n* = 3		2.02 m (0.03), *n* = 3	53
Bridgett and Linthorne [61]	Long jump	Field	8.30 m, *n* = 1			67
Hay et al. [16]	Long jump	Competition	8.02 m (0.38), *n* = 12			67
Linthorne et al. [60]	Long jump	Field	8.30 m, *n* = 1			60
Pavlović et al. [59]	Long jump	Competition	8.24 m (0.19), *n* = 8		6.80 m (0.16), *n* = 8	53
Čoh et al. [22]	Triple jump	Laboratory			15.03, *n* = 1	73
Miller and Hay [62]	Triple jump	Competition	17.70 m (0.21), *n* = 4			60
Panoutsakopoulos and Kollias [65]	Triple jump	Competition			14.66 m (0.43), *n* = 9	67
Pavlović [18]	Triple jump	Competition	17.37 m (0.29), *n* = 16			53
Pavlović and de Oliveria [17]	Triple jump	Competition			14.54 m (0.26), *n* = 16	53
Yu. [64]	Triple jump	Competition	17.60 m (0.68), *n* = 5		14.41 m (0.20), *n* = 2	73
Yu and Hay [63]	Triple jump	Competition	17.43 m (0.45), *n* = 4			73
Graubner and Nixdorf [33]	100/110-m hurdles, 400- m hurdles, 100-m sprint	Competition	110 mH: 13.32 s (0.16), *n* = 8 400 mH: 48.32 s (0.28), *n* = 7 100 m: 9.92 s (0.23), *n* = 8		100 mH: 12.65 s (0.12), *n* = 7 400 mH: 53.74 s (0.89), *n* = 8 100 m: 10.95 s (0.15), *n* = 8	60
Müller and Honinicl [31]	100/110 m hurdles, 100–400 m sprint, high jump, long jump, triple jump	Competition	110 mH: 13.21 s (0.20), *n* = 7 100 m: 9.96 s (0.09), *n* = 6 200 m: 20.29 s (0.13), *n* = 8 400 m: 44.52 s (0.32), *n* = 8 High jump: 2.34 m (0.02), *n* = 6 Long jump: 8.12 m (0.20), *n* = 8 Triple jump: 17.42 m (0.30), *n* = 8		100 mH: 12.72 s (0.16), *n* = 7 100 m: 11.07 s (0.19), *n* = 6 200 m: 22.54 s (0.17), *n* = 8 400 m: 50.31 s (0.56), *n* = 8 Long jump: 6.81 m (0.14), *n* = 9 Triple jump: 14.57 m (0.44), *n* = 8	47

*m* metres, *mH* hurdles, *n* number of athletes, *N/A* not available, *s* seconds, *SD* standard deviation

### Results by Event Group in Track-Based Events

Table 3 details the number of athletes, in track-based events, assessed across characteristics identified in this review. Characteristics are grouped as anthropometrical, biomechanical or performance outcome measures. The subsequent sections report the specific results and compare between medallists and non-medallists (where possible) for each individual speed/power-based track event.

**Table 3:**
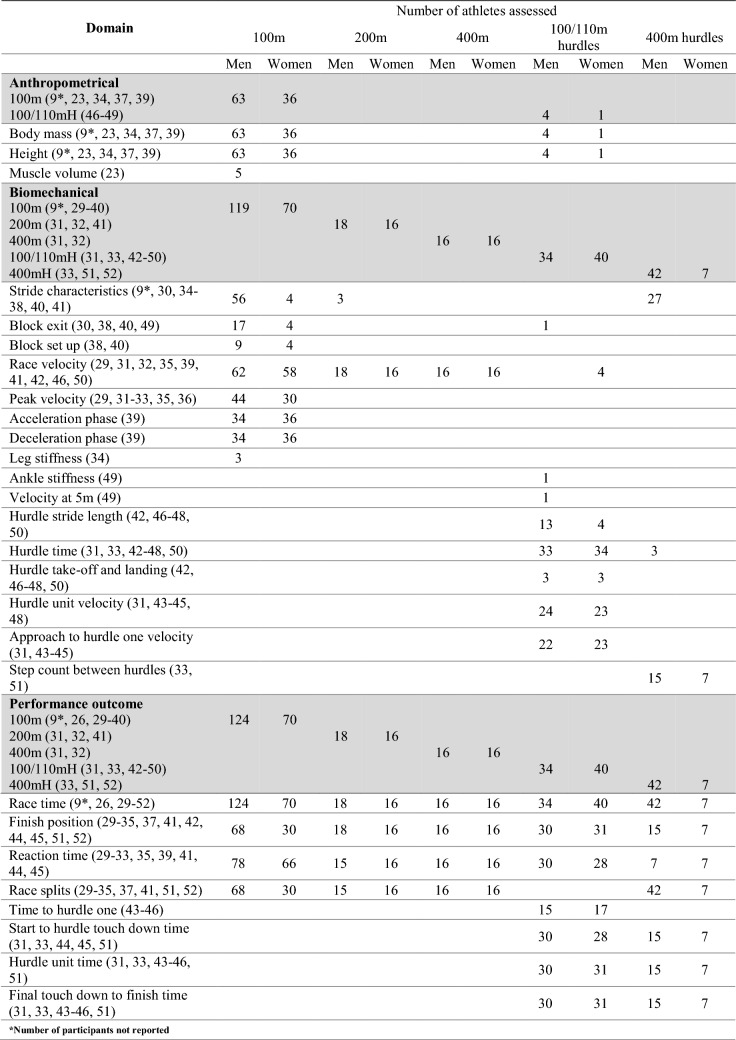
Number of athletes assessed across identified characteristics in speed/power-based track events

^a^Number of participants not reported

#### 100-m Sprint

Data for the 100-m sprint were extracted from the competition-based (*n* = 12) and laboratory-based (*n* = 2) literature (Table 2). A comparison between medallists and non-medallists during competition is available in Table 4. Seven competition-based papers reported individual analysis of the 100-m sprint for a total of 33 men (12 medallists and 21 non-medallists) and 24 women (10 medallists and 14 non-medallists) [29–35]. Compared with non-medallists, both the men and women medallists reached a greater peak velocity (+ 0.46 and + 0.22 m/s respectively) and while men achieved peak velocity at a later stage of the race (60–70-m section), there was no difference for women. Male medallists were faster across 40–50 m (0.03 s), 70–80 m (0.03 s), 80–90 m (0.03 s) and 90–100 m (0.03 s) whereas non-medallists were faster from 0 to 10 m (0.03 s). Female medallists were faster across the 30–40 m (0.03 s), 70–80 m (0.03 s), 80–90 m (0.03 s) and 90–100 m (0.06 s) intervals, compared with non-medallists.

**Table 4:** Summary of 100-m sprint competition analysis for men and women, grouped as medallists and non-medallists [29–35]

	Men					Women				
Variable	Medallist		Non-medallist			Medallist		Non-medallist		
	*n*	Mean (SD)	*n*	Mean (SD)	Difference^a^	*n*	Mean (SD)	*n*	Mean (SD)	Difference^a^
Finish time (s)	12	9.86 (0.13)	21	10.11 (0.14)	− 0.25	10	10.87 (0.13)	14	11.08 (0.11)	− 0.21
Reaction time (s)	12	0.14 (0.02)	21	0.16 (0.03)	− 0.02	10	0.15 (0.03)	14	0.16 (0.04)	− 0.01
Mean velocity (m/s)	5	10.07 (0.1)	9	9.87 (0.12)	0.20	3	9.25 (0.11)	5	9.08 (0.08)	0.17
Peak velocity (m/s)	7	11.97 (0.22)	11	11.51 (0.17)	0.46	7	10.72 (0.13)	10	10.50 (0.10)	0.22
Section of peak velocity (m)	7	60–70	11	50–60		7	50–60	10	50–60	
Interval times (s)										
0−10	10	1.82 (0.07)	11	1.78 (0.06)	0.03	4	1.86 (0.03)	6	1.85 (0.02)	0.01
10−20	10	1.03 (0.03)	11	1.04 (0.02)	− 0.01	4	1.12 (0.02)	6	1.12 (0.01)	0.00
20−30	10	0.92 (0.01)	11	0.93 (0.01)	− 0.01	4	1.01 (0.02)	6	1.01 (0.01)	0.00
30−40	10	0.88 (0.01)	11	0.89 (0.01)	− 0.02	4	0.96 (0.01)	6	0.99 (0.01)	− 0.03
40−50	10	0.85 (0.01)	11	0.88 (0.01)	− 0.03	4	0.94 (0.01)	6	0.96 (0.01)	− 0.02
50−60	10	0.85 (0.02)	11	0.87 (0.01)	− 0.02	4	0.94 (0.01)	6	0.96 (0.01)	− 0.02
60−70	10	0.85 (0.02)	11	0.87 (0.01)	− 0.02	4	0.95 (0.01)	6	0.97 (0.02)	− 0.02
70−80	10	0.85 (0.02)	11	0.88 (0.01)	− 0.03	4	0.96 (0.01)	6	0.99 (0.01)	− 0.03
80−90	10	0.86 (0.02)	11	0.90 (0.01)	− 0.03	4	0.98 (0.01)	6	1.01 (0.02)	− 0.03
90−100	10	0.87 (0.02)	11	0.90 (0.02)	− 0.03	4	0.99 (0.02)	6	1.05 (0.07)	− 0.06
Interval velocity (m/s)										
0−10	6	5.73 (0.06)	5	5.67 (0.07)	0.07	3	5.40 (0.10)	5	5.42 (0.02)	− 0.02
10−20	6	9.85 (0.12)	5	9.76 (0.08)	0.08	3	9.04 (0.05)	5	8.98 (0.09)	0.06
20−30	6	10.95 (0.12)	5	10.80 (0.1)	0.15	3	9.90 (0.20)	5	9.92 (0.14)	− 0.02
30−40	6	11.40 (0.21)	5	11.11 (0.09)	0.29	3	10.49 (0.06)	5	10.12 (0.11)	0.37
40−50	6	11.78 (0.17)	5	11.24 (0.09)	0.55	3	10.68 (0.06)	5	10.37 (0.13)	0.30
50−60	6	11.95 (0.19)	5	11.55 (0.12)	0.40	3	10.68 (0.17)	5	10.40 (0.09)	0.28
60−70	6	11.85 (0.30)	5	11.47 (0.11)	0.38	3	10.57 (0.06)	5	10.27 (0.18)	0.30
70−80	6	11.78 (0.33)	5	11.29 (0.07)	0.49	3	10.46 (0.06)	5	10.14 (0.15)	0.31
80−90	6	11.72 (0.32)	5	11.17 (0.24)	0.56	3	10.27 (0.06)	5	9.96 (0.11)	0.31
90−100	6	11.5 (0.33)	5	11.09 (0.24)	0.41	3	10.17 (0.16)	5	9.69 (0.12)	0.48
Mean stride frequency (Hz)	3	4.51 (0.38)	4	4.46 (0.24)	0.05					
Mean stride length (m)	3	2.24 (0.21)	4	2.20 (0.13)	0.04					

*Hz* Hertz, *m* metres, *m/s* metres per second, *n* number of athletes, *s* seconds, *SD* standard deviation

^a^Difference = medallist—non-medallist

A total of five competition-based papers reported mean results, for men and women separately, for groups of between 4 and 36 athletes [9, 35–39]. While some studies did not enable a comparison between medallists and non-medallists, athletes in these studies enhanced understanding of the profile of the elite athlete. Using magnetic resonance imaging, Miller et al. [23] characterised their elite male sprint group as having a body mass of 86.4 ± 6.7 kg, 8.3 ± 1.2% body fat, waist-to-glute ratio of 0.84 ± 0.05 and full body relative muscle volume of 131.3 ± 6.8 cm^3^/kg. Colyer et al. [40] focused on the block start with elite athletes achieving a horizontal and vertical block exit velocity of 3.36 ± 0.13 and 0.58 ± 0.06 m/s respectively, centre of mass projection angle of 9.8 ± 0.8 º and relative horizontal impulse of 0.247 ± 0.015 and 0.106 ± 0.017 on the front and rear blocks, respectively.

#### 200-m Sprint

Data for the 200-m sprint were extracted from the competition-based (*n* = 3) literature only (Table 2). A comparison between medallists and non-medallists during competition is available in Table 5. All competition-based papers reported individual analysis of the 200 m for a total of 13 men (five medallists and eight non-medallists) and 15 women (six medallists and nine non-medallists) [31, 32, 41]. Compared with non-medallists, male medallists were faster across 50–100 m (0.1 s) and 150–200 m (0.11 s) while female medallists were faster across 0–50 m (0.13 s) and 50–100 m (0.12 s). The difference in the 150–200-m split was similar for both the women’s medallists and non-medallists. In the 1984 men’s Olympic final, medallists achieved a greater stride length and frequency at both 125 m (length: 2.38 ± 0.00 m, frequency: 4.24 ± 0.09 Hz) and 180 m (length: 2.49 ± 0.01 m, frequency 4.26 ± 0.13 Hz) than the eighth-place finisher (125 m length: 2.38 m and frequency: 4.01 Hz, 180 m length: 2.38 m and frequency: 4.17 Hz) [41].

**Table 5:** Summary of the 200-m sprint competition analysis for men and women, grouped as medallists and non-medallists [31, 32, 41]

			Men								Women				
Variable	Medallist		Non-medallist					Medallist			Non-medallist				
	*n*	Mean (SD)		*n*	Mean (SD)		Difference^a^		*n*	Mean (SD)		*n*	Mean (SD)		Difference^a^
Finish time (s)	5	20.06 (0.12)		8	20.36 (0.09)		− 0.31		6	22.23 (0.23)		9	22.54 (0.19)		− 0.31
Reaction time (s)	5	0.14 (0.02)		8	0.14 (0.02)		0.00		6	0.15 (0.03)		9	0.15 (0.01)		0.00
Interval time (s)															
0–50 m	5	5.76 (0.09)		8	5.80 (0.14)		− 0.04		6	6.13 (0.14)		9	6.26 (0.07)		− 0.13
50– 100 m	5	4.49 (0.08)		8	4.59 (0.07)		− 0.10		6	4.99 (0.04)		9	5.11 (0.07)		− 0.12
100– 150 m	5	4.74 (0.06)		8	4.82 (0.07)		− 0.08		6	5.26 (0.07)		9	5.35 (0.03)		− 0.09
150–200 m	5	5.00 (0.07)		8	5.11 (0.09)		− 0.11		6	5.77 (0.17)		9	5.75 (0.07)		0.02
Interval velocity (m/s)															
0–50 m	5	8.68 (0.14)		8	8.63 (0.21)		0.05		6	8.16 (0.19)		9	7.99 (0.08)		0.17
50–100 m	5	11.08 (0.18)		8	10.89 (0.17)		0.19		6	10.02 (0.07)		9	9.79 (0.13)		0.23
100–150 m	5	10.54 (0.13)		8	10.43 (0.08)		0.11		6	9.50 (0.13)		9	9.34 (0.06)		0.16
150–200 m	5	10.02 (0.14)		8	9.79 (0.17)		0.23		6	8.68 (0.25)		9	8.70 (0.11)		− 0.02

*m* metres, *m/s* metres per second, *n* number of athletes, *s* seconds, *SD* standard deviation

^a^Difference = medallist—non-medallist

#### 400-m Sprint

Data for the 400-m sprint were extracted from the competition-based (*n* = 2) literature only (Table 2). A comparison between medallists and non-medallists during competition is available in Table 6. Both competition-based papers reported an individual competition analysis in the 400 m that resulted in a total of ten men (five medallists and five non-medallists) and 13 women (five medallists and eight non-medallists) [31, 32]. Male medallists were faster across 250–300 m (0.18 s), 300–350 m (0.22 s) and 350–400 m (0.29 s) while non-medallists were faster across 0–50 m (0.11 s). Compared with non-medallists, female medallists were faster across 200–250 m (0.13 s), 250–300 m (0.15 s), 300–350 m (0.14 s) and 350–400 m (0.29 s).

**Table 6:** Summary of 400-m sprint competition analysis for men and women, grouped as medallists and non-medallists [31, 32]

	Men					Women				
Variable	Medallist		Non-medallist			Medallist		Non-medallist		
	*n*	Mean (SD)	*n*	Mean (SD)	Difference^a^	*n*	Mean (SD)	*n*	Mean (SD)	Difference^a^
Finish time (s)	5	44.11 (0.52)	5	44.79 (0.32)	− 0.68	5	49.8 (0.11)	8	50.66 (0.36)	− 0.85
Reaction time (s)	5	0.17 (0.03)	5	0.18 (0.03)	0.00	5	0.18 (0.02)	8	0.17 (0.02)	0.00
Interval time (s)										
0–50 m	5	6.13 (0.10)	5	6.03 (0.18)	0.11	5	6.63 (0.11)	8	6.66 (0.13)	− 0.03
50–100 m	5	4.98 (0.05)	5	4.96 (0.05)	0.01	5	5.57 (0.05)	8	5.63 (0.08)	− 0.05
100–150 m	5	5.01 (0.07)	5	5.02 (0.06)	0.00	5	5.66 (0.08)	8	5.67 (0.15)	− 0.01
150–200 m	5	5.14 (0.10)	5	5.18 (0.07)	− 0.04	5	5.91 (0.04)	8	5.96 (0.08)	− 0.05
200–250 m	5	5.34 (0.11)	5	5.41 (0.04)	− 0.07	5	6.10 (0.05)	8	6.23 (0.09)	− 0.13
250–300 m	5	5.44 (0.13)	5	5.62 (0.09)	− 0.18	5	6.21 (0.10)	8	6.36 (0.13)	− 0.15
300–350 m	5	5.76 (0.14)	5	5.98 (0.10)	− 0.22	5	6.56 (0.07)	8	6.70 (0.13)	− 0.14
350–400 m	5	6.23 (0.17)	5	6.52 (0.19)	− 0.29	5	7.11 (0.08)	8	7.40 (0.26)	− 0.29
Interval velocity (m/s)										
0–50 m	5	8.15 (0.13)	5	8.30 (0.26)	− 0.15	5	7.54 (0.13)	8	7.51 (0.14)	0.03
50–100 m	5	10.04 (0.10)	5	10.07 (0.10)	− 0.03	5	8.97 (0.08)	8	8.89 (0.12)	0.09
100–150 m	5	9.97 (0.14)	5	9.97 (0.11)	0.00	5	8.83 (0.12)	8	8.82 (0.23)	0.01
150–200 m	5	9.72 (0.20)	5	9.70 (0.20)	0.02	5	8.46 (0.06)	8	8.40 (0.13)	0.06
200–250 m	5	9.37 (0.20)	5	9.25 (0.06)	0.12	5	8.20 (0.07)	8	8.03 (0.11)	0.17
250–300 m	5	9.01 (0.44)	5	8.57 (0.39)	0.44	5	8.06 (0.13)	8	7.86 (0.17)	0.19
300–350 m	5	8.69 (0.21)	5	8.37 (0.14)	0.32	5	7.63 (0.08)	8	7.47 (0.14)	0.16
350–400 m	5	8.03 (0.22)	5	7.67 (0.22)	0.36	5	7.03 (0.08)	8	6.77 (0.23)	0.27

*m* metres, *m/s* metres per second, *n* number of athletes, *s* seconds, *SD* standard deviation

^a^Difference = medallist—non-medallist

#### 100/110-m Hurdles

Data for the 100/110-m hurdles were extracted from the competition-based (*n* = 9) and field-based (*n* = 2) literature (Table 2). A comparison between medallists and non-medallists during competition is available in Table 7. Six competition-based papers reported an individual analysis of the 100/110-m hurdles [31, 33, 42–45]. There were a total of 26 men (10 medallists and 16 non-medallists) and 25 women (10 medallists and 15 non-medallists). The average finish time for medallists was 0.31 and 0.24 s faster for men and women, respectively. The greatest difference in hurdle unit time for men hurdles occurred from hurdles 5–6 (0.03 s), 8–9 (0.03 s) and 9–10 (0.04 s), with medallists covering the distance faster each time. Hurdle clearance time was similar between medallists and non-medallists for all hurdles expect hurdle six, where medallists were 0.02 s faster. The greatest difference in hurdle unit time for women was hurdles 8–9 (0.03 s) and 9–10 (0.04 s) with medallists covering the distance faster each time. Hurdle clearance times were similar for all women.

**Table 7:** Summary of 100/110-m hurdles competition analysis for men and women, grouped as medallists and non-medallists [31, 33, 42–45]

	Men (110 m)					Women (100 m)				
Variable	Medallist		Non-medallist			Medallist		Non-medallist		
	*n*	Mean (SD)	*n*	mean (SD)	Difference^a^	*n*	mean (SD)	*n*	Mean (SD)	Difference^a^
Finish time (s)	10	13.09 (0.12)	16	13.40 (0.16)	− 0.31	10	12.53 (0.1)	15	12.76 (0.1)	− 0.24
Reaction time (s)	10	0.15 (0.02)	16	0.15 (0.02)	0.00	9	0.15 (0.02)	14	0.16 (0.03)	0.00
Time from start to touchdown after hurdle (s)										
1	10	2.55 (0.04)	16	2.60 (0.05)	− 0.05	9	2.55 (0.03)	14	2.59 (0.03)	− 0.04
2	7	3.59 (0.04)	12	3.66 (0.06)	− 0.08	6	3.56 (0.04)	10	3.60 (0.04)	− 0.04
3	7	4.59 (0.06)	12	4.69 (0.07)	− 0.10	6	4.54 (0.05)	10	4.59 (0.05)	− 0.06
4	7	5.59 (0.06)	12	5.72 (0.08)	− 0.13	6	5.51 (0.05)	10	5.57 (0.07)	− 0.06
5	7	6.59 (0.07)	12	6.74 (0.09)	− 0.15	6	6.50 (0.06)	10	6.55 (0.06)	− 0.05
6	7	7.59 (0.08)	12	7.77 (0.10)	− 0.18	6	7.47 (0.07)	10	7.52 (0.07)	− 0.06
7	7	8.61 (0.08)	12	8.81 (0.11)	− 0.20	6	8.44 (0.07)	10	8.51 (0.07)	− 0.07
8	7	9.64 (0.10)	12	9.86 (0.12)	− 0.22	6	9.43 (0.08)	10	9.51 (0.09)	− 0.08
9	7	10.68 (0.10)	12	10.92 (0.13)	− 0.24	6	10.43 (0.10)	10	10.54 (0.08)	− 0.11
10	7	11.72 (0.11)	12	12.01 (0.15)	− 0.28	6	11.44 (0.11)	10	11.59 (0.08)	− 0.15
Approach to hurdle 1 time (s)	6	2.38 (0.04)	8	2.43 (0.04)	− 0.05	7	2.40 (0.07)	9	2.44 (0.07)	− 0.03
Hurdle unit time (s)										
1–2	10	1.04 (0.02)	16	1.06 (0.02)	− 0.02	10	1.02 (0.02)	15	1.01 (0.02)	0.01
2–3	10	1.00 (0.02)	16	1.02 (0.02)	− 0.02	10	0.98 (0.02)	15	1.00 (0.02)	− 0.02
3–4	10	1.00 (0.01)	16	1.02 (0.01)	– 0.02	10	0.97 (0.01)	15	0.98 (0.02)	0.00
4–5	10	1.00 (0.02)	16	1.02 (0.02)	− 0.02	10	0.98 (0.02)	15	0.98 (0.02)	0.00
5–6	10	1.01 (0.01)	16	1.04 (0.03)	− 0.03	10	0.97 (0.02)	15	0.98 (0.02)	− 0.01
6–7	10	1.01 (0.02)	16	1.03 (0.02)	− 0.02	10	0.97 (0.02)	15	0.99 (0.02)	− 0.02
7–8	10	1.03 (0.02)	16	1.05 (0.02)	− 0.02	10	0.98 (0.01)	15	1.01 (0.02)	− 0.02
8–9	10	1.04 (0.01)	16	1.06 (0.02)	− 0.03	10	0.99 (0.02)	15	1.03 (0.02)	− 0.03
9–10	10	1.04 (0.02)	16	1.09 (0.03)	− 0.04	10	1.02 (0.02)	15	1.05 (0.02)	– 0.04
Run to finish line time (s)	10	1.36 (0.03)	16	1.41 (0.05)	− 0.05	10	1.10 (0.03)	15	1.14 (0.05)	– 0.04
Hurdle clearance time (s)										
1	10	0.34 (0.01)	12	0.34 (0.03)	− 0.01	10	0.32 (0.03)	13	0.31 (0.02)	0.01
2	10	0.34 (0.01)	12	0.35 (0.03)	− 0.01	10	0.31 (0.03)	13	0.30 (0.02)	0.01
3	10	0.33 (0.02)	12	0.33 (0.03)	0.00	10	0.31 (0.02)	13	0.31 (0.02)	0.00
4	10	0.33 (0.02)	12	0.34 (0.03)	− 0.01	10	0.3 (0.02)	13	0.29 (0.02)	0.00
5	10	0.33 (0.02)	12	0.33 (0.03)	0.00	10	0.31 (0.02)	13	0.30 (0.01)	0.00
6	10	0.33 (0.01)	12	0.34 (0.04)	− 0.02	10	0.31 (0.02)	13	0.30 (0.03)	0.01
7	10	0.33 (0.01)	12	0.34 (0.02)	− 0.01	10	0.29 (0.02)	13	0.30 (0.02)	0.00
8	10	0.35 (0.03)	12	0.34 (0.02)	0.00	10	0.30 (0.02)	13	0.30 (0.02)	0.00
9	10	0.34 (0.01)	12	0.34 (0.03)	0.00	10	0.32 (0.03)	13	0.31 (0.02)	0.00
10	10	0.34 (0.01)	12	0.35 (0.03)	− 0.01	10	0.32 (0.02)	13	0.32 (0.02)	0.00
Approach to hurdle 1 velocity (m/s)	9	5.86 (0.1)	12	5.76 (0.1)	0.1	10	5.77 (0.23)	13	5.69 (0.22)	0.08
Hurdle unit velocity (m/s)										
1–2	9	8.78 (0.16)	12	8.59 (0.2)	0.19	10	8.37 (0.19)	13	8.4 (0.22)	− 0.03
2–3	9	9.11 (0.19)	12	8.96 (0.17)	0.15	10	8.67 (0.18)	13	8.5 (0.13)	0.17
3–4	9	9.13 (0.12)	12	8.98 (0.13)	0.15	10	8.74 (0.11)	13	8.69 (0.21)	0.04
4–5	9	9.03 (0.34)	12	8.99 (0.18)	0.04	10	8.71 (0.17)	13	8.61 (0.14)	0.1
5–6	9	9.1 (0.13)	12	8.85 (0.23)	0.25	10	8.75 (0.16)	13	8.68 (0.18)	0.07
6–7	9	9.04 (0.21)	12	8.87 (0.17)	0.18	10	8.76 (0.13)	13	8.54 (0.17)	0.21
7–8	9	8.87 (0.19)	12	8.74 (0.22)	0.13	10	8.64 (0.12)	13	8.4 (0.26)	0.24
8–9	9	8.83 (0.09)	12	8.59 (0.14)	0.24	10	8.55 (0.17)	13	8.29 (0.16)	0.26
9–10	9	8.76 (0.14)	12	8.44 (0.23)	0.33	10	8.37 (0.15)	13	8.08 (0.19)	0.29
Approach to finish line velocity (m/s)	9	9.42 (0.2)	12	9.09 (0.37)	0.34	10	8.5 (0.22)	13	8.16 (0.38)	0.34

*m/s* metres per second, *n* number of athletes, s seconds, *SD* standard deviation

^a^Difference = medallist – non-medallist

Three competition-based papers analysed the clearance over the fourth and fifth hurdle only [46–48]. Charalambous et al. [49] reported characteristics such as mean first stance contact time (0.21 ± 0.01 s), velocity at 5 m (6.51 ± 0.12 m/s) and ankle stiffness (5.93 ± 0.75 Nm/º) of an elite male across eight block start trials.

#### 400-m Hurdles

Data for the 400-m hurdles were extracted from the competition-based (*n* = 3) literature only (Table 2). A comparison between medallists and non-medallists during competition is available in Table 8. Two competition-based papers reported an individual competition analysis for a total of 15 men (six medallists and nine non-medallists) and eight women (three medallists and five non-medallists) [33, 51]. Medallists in the 400-m hurdles were 0.67 and 1.40 s faster than the non-medallists for men and women, respectively. For men, the greatest difference in hurdle unit time occurred between hurdles 6–7 (0.08 s), 7–8 (0.08 s) and 9–10 (0.13 s) and from hurdle 10 touchdown to finish line (0.26 s), with medallists covering the distance faster each time. The greatest difference in hurdle unit time for women occurred between hurdles 6–7 (0.18 s), 7–8 (0.19 s), 8–9 (0.22 s) and 9–10 (0.18 s) and from hurdle 10 touchdown to finish line (0.25 s), with medallists again faster across each interval.

**Table 8:** Summary of 400-m hurdles competition analysis for men and women, grouped as medallists and non-medallists [33, 51]

	Men					Women				
Variable	Medallist		Non-medallist			Medallist		Non-medallist		
	*n*	Mean (SD)	*n*	Mean (SD)	Difference^a^	*n*	Mean (SD)	*n*	Mean (SD)	Difference^a^
Finish time (s)	6	47.85 (0.29)	9	48.51 (0.30)	− 0.67	3	52.86 (0.40)	5	54.26 (0.63)	− 1.40
Reaction time (s)	3	0.17 (0.02)	4	0.17 (0.02)	0.00	3	0.16 (0.02)	5	0.18 (0.03)	− 0.02
Time from start to touchdown after hurdle (s)										
1	6	5.91 (0.08)	9	6.04 (0.13)	− 0.13	3	6.22 (0.05)	5	6.41 (0.17)	− 0.19
2	6	9.66 (0.06)	9	9.8 (0.16)	− 0.14	3	10.15 (0.05)	5	10.44 (0.20)	− 0.29
3	6	13.46 (0.12)	9	13.6 (0.19)	− 0.13	3	14.28 (0.05)	5	14.57 (0.22)	− 0.28
4	6	17.37 (0.21)	9	17.44 (0.20)	− 0.07	3	18.56 (0.05)	5	18.84 (0.24)	− 0.29
5	6	21.37 (0.30)	9	21.42 (0.25)	− 0.06	3	22.98 (0.08)	5	23.24 (0.35)	− 0.26
6	6	25.44 (0.34)	9	25.51 (0.27)	− 0.07	3	27.48 (0.03)	5	27.86 (0.44)	− 0.38
7	6	29.58 (0.38)	9	29.73 (0.29)	− 0.15	3	32.09 (0.08)	5	32.65 (0.49)	− 0.56
8	6	33.85 (0.39)	9	34.04 (0.27)	− 0.19	3	36.81 (0.16)	5	37.56 (0.56)	− 0.75
9	6	38.27 (0.37)	9	38.55 (0.29)	− 0.28	3	41.76 (0.22)	5	42.74 (0.63)	− 0.97
10	6	42.75 (0.35)	9	43.27 (0.37)	− 0.52	3	46.94 (0.29)	5	48.1 (0.71)	− 1.16
Hurdle unit time (s)										
1–2	6	3.75 (0.07)	9	3.76 (0.07)	− 0.01	3	3.94 (0.02)	5	4.03 (0.05)	− 0.10
2–3	6	3.80 (0.09)	9	3.80 (0.06)	0.01	3	4.13 (0.04)	5	4.13 (0.06)	0.00
3–4	6	3.91 (0.11)	9	3.84 (0.06)	0.06	3	4.27 (0.08)	5	4.28 (0.07)	0.00
4–5	6	4.00 (0.11)	9	3.99 (0.06)	0.01	3	4.42 (0.03)	5	4.40 (0.14)	0.02
5–6	6	4.07 (0.08)	9	4.09 (0.06)	− 0.02	3	4.50 (0.06)	5	4.62 (0.13)	− 0.12
6–7	6	4.14 (0.10)	9	4.22 (0.06)	− 0.08	3	4.61 (0.10)	5	4.79 (0.12)	− 0.18
7–8	6	4.27 (0.08)	9	4.35 (0.05)	− 0.08	3	4.72 (0.09)	5	4.91 (0.12)	− 0.19
8–9	6	4.42 (0.06)	9	4.47 (0.06)	− 0.06	3	4.95 (0.16)	5	5.17 (0.16)	− 0.22
9–10	6	4.48 (0.05)	9	4.61 (0.07)	− 0.13	3	5.18 (0.12)	5	5.36 (0.19)	− 0.18
Run to finish line time (s)	6	5.1 (0.11)	9	5.36 (0.11)	− 0.26	3	5.92 (0.11)	5	6.16 (0.28)	− 0.25
Hurdle step count (nH)										
1–2	6	13.33 (0.52)	9	13.33 (0.50)	0.00	3	14.67 (0.58)	5	14.6 (0.55)	0.07
2–3	6	13.33 (0.52)	9	13.33 (0.50)	0.00	3	14.67 (0.58)	5	14.6 (0.55)	0.07
3–4	6	13.33 (0.52)	9	13.33 (0.50)	0.00	3	14.67 (0.58)	5	14.8 (0.45)	− 0.13
4–5	6	13.33 (0.52)	9	13.44 (0.53)	− 0.11	3	14.67 (0.58)	5	14.8 (0.84)	− 0.13
5–6	6	13.50 (0.84)	9	13.44 (0.53)	0.06	3	15.00 (0.00)	5	15.2 (0.45)	− 0.20
6–7	6	13.50 (0.84)	9	13.89 (0.6)	− 0.39	3	15.33 (0.58)	5	15.6 (0.55)	− 0.27
7–8	6	13.83 (0.98)	9	14.22 (0.67)	− 0.39	3	15.33 (0.58)	5	15.6 (0.55)	− 0.27
8–9	6	14.00 (0.89)	9	14.33 (0.50)	− 0.33	3	16.33 (0.58)	5	16.4 (0.89)	− 0.07
9–10	6	14.17 (0.75)	9	14.56 (0.53)	− 0.39	3	16.67 (0.58)	5	16.8 (1.10)	− 0.13

*n* number of athletes, *n* number of steps, *s* seconds, *SD* standard deviation

^a^Difference = medallist—non-medallist

One competition-based paper compared a male cohort of 13 world class and 14 elite hurdlers across nine variables [52]. Similarities between groups emerged in step length, both overall and when broken down to the examine the first 200 and second 200 m. Compared with the elite group, the world class group attained a greater step frequency in the first 200 m (world class: 3.75 ± 0.12 Hz, elite: 3.6 ± 0.11 Hz) and overall (world class: 3.59 ± 0.14 Hz, elite: 3.50 ± 0.10 Hz).

### Results by Event Group in Field-Based Events

Table 9 details the number of athletes, in field-based events, assessed across characteristics identified in this review. Characteristics are grouped as anthropometrical, biomechanical or performance outcome measures. The subsequent sections report the specific results and compare between medallists and non-medallists (where possible) for each individual speed/power-based field event.

**Table 9:**
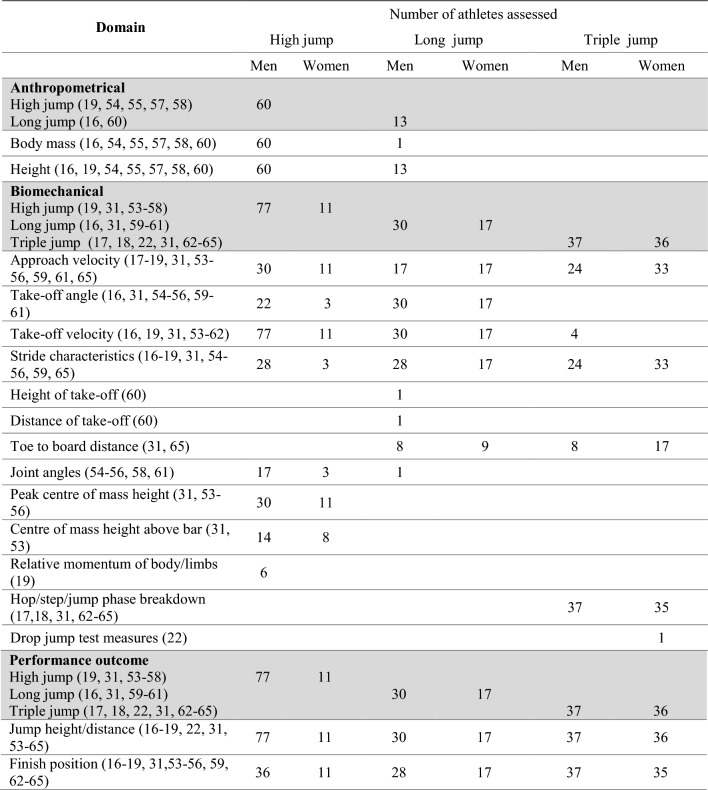
Number of athletes assessed across identified characteristics in speed/power-based field events

#### High Jump

Data for the high jump were extracted from the competition-based (*n* = 7) and field-based (*n* = 1) literature (Table 2). A comparison between medallists and non-medallists during competition is available in Table 10. Six papers reported an individual competition analysis of high jump for a total of 30 men (14 medallists and 16 non-medallists) and 11 women (six medallists and five non-medallists) [19, 31, 53–56]. Notable differences for men occurred in medallists attaining a greater vertical velocity at touchdown prior to (0.15 m/s) and at take-off (0.17 m/s) than non-medallists. Similarities between medallists and non-medallists for men occurred in stature (0 m), take-off angle (0.15º) and length of the last stride before take-off (0.05 m). Compared with non-medallists, notable differences for women occurred in medallists attaining a greater horizontal velocity at touchdown prior to take-off (0.47 m/s) and vertical velocity at take-off (0.4 m/s).

**Table 10:** Summary of high jump competition analysis for men and women, grouped as medallists and non-medallists [19, 31, 53–56]

	Men					Women				
Variable	Medallist		Non-medallist			Medallist		Non-medallist		
	*n*	Mean (SD)	*n*	Mean (SD)	Difference^a^	*n*	Mean (SD)	*n*	Mean (SD)	Difference^a^
Jump height (m)	14	2.33 (0.03)	16	2.29 (0.04)	0.04	6	2.01 (0.04)	5	1.93 (0.02)	0.08
Stature (m)	8	1.91 (0.05)	9	1.91 (0.05)	0					
Body mass (kg)	8	75.25 (9.36)	9	74.33 (4.39)	0.92					
Peak centre of mass height (m)	11	2.44 (0.09)	13	2.34 (0.04)	0.1	6	2.11 (0.08)	5	1.98 (0.04)	0.13
Centre of mass height above bar (m)	5	0.07 (0.04)	7	0.05 (0.03)	0.03					
Take-off angle (deg)	9	50.04 (2.11)	9	50.19 (1.94)	− 0.15					
Velocity at touchdown prior to take-off (m/s)										
Horizontal	10	7.51 (0.38)	13	7.45 (0.47)	0.06	3	6.87 (0.32)	5	6.4 (0.55)	0.47
Vertical	8	0.11 (0.54)	9	– 0.04 (0.46)	0.15					
Velocity at take-off (m/s)										
Horizontal	9	4.01 (0.28)	9	4.11 (0.51)	− 0.1					
Vertical	14	4.5 (0.19)	16	4.34 (0.19)	0.17	6	3.92 (0.26)	5	3.52 (0.16)	0.4
Foot contact time at take-off (s)	9	0.18 (0.02)	9	0.17 (0.02)	0.01					
Knee angle at take-off (deg)										
Contact	6	165.49 (9.05)	6	168.22 (4.64)	− 2.72					
Extension	4	172.79 (3.91)	6	173.37 (4.29)	− 0.58					
Length of last stride before take-off (m)	6	2.03 (0.11)	9	2.08 (0.12)	− 0.05					
Lead leg velocity (m/s)										
Peak	3	6.3 (0.26)	5	6.38 (0.29)	− 0.08	3	6.37 (0.4)	5	5.78 (0.46)	0.59
Take-off	3	5.43 (0.57)	5	5.28 (0.49)	0.15	3	3.2 (0.6)	5	4.4 (0.49)	− 1.2
Deceleration after take-off	3	− 0.87 (0.38)	5	− 1.1 (0.61)	0.23	3	− 3.17 (0.91)	5	− 1.38 (0.62)	− 1.79
Arm velocity (m/s)										
Peak	3	7.77 (0.21)	5	7.4 (1.19)	0.37	3	5.03 (2.9)	5	5.96 (1.23)	− 0.93
Take-off	3	7.2 (0.62)	5	7 (1.03)	0.2	3	4.63 (2.7)	5	5.16 (1.37)	− 0.53
Deceleration after take-off	3	− 0.57 (0.55)	5	− 0.56 (0.34)	– 0.01	3	− 0.4 (0.26)	5	− 0.8 (0.57)	0.4

*deg* degree, *kg* kilograms, *m* metres, *m/s* metres per second,* n* = number of athletes, *s* seconds, *SD* standard deviation

^a^Difference = medallist—non-medallist

One competition-based paper reported the mean results from a group of 40 men [57]. Six variables were used to characterise this group and included height (1.90 ± 0.06 m), body mass (78 ± 5 kg) horizontal velocity at take-off (7.45 ± 0.34 m/s), vertical velocity at take-off (4.31 ± 0.14 m/s), maximum vertical velocity of the arm relative to the trunk during the take-off phase (8.68 ± 1.3 m/s) and height of centre of mass at take-off relative to standing height (49.1 ± 1.9%). One field-based paper characterised the take-off position of an elite male jumper [58]. Reported variables included height (1.96 m) and body mass (78.7 kg) and mean (of 16 trials) approach velocity (6.60 ± 0.45 m/s), plant angle (34.1 ± 1.8°) and knee angle at touchdown (170.3 ± 5.6°).

#### Long Jump

Data for the long jump were extracted from the competition-based (*n* = 3) and field-based literature (*n* = 2) [Table 2]. A comparison between medallists and non-medallists during competition is presented in Table 11. Three papers reported an individual competition analysis in long jump for a total of 24 men (six medallists and 15 non-medallists) and 17 women (six medallists and 11 non-medallists) [16, 31, 59]. Notable differences for men occurred with medallists attaining a greater mean approach velocity from 11–6 m (0.39 m/s) and 6–1 m (0.29 m/s) and horizontal velocity at take-off (0.29 m/s). Similarities between the men’s medallists and non-medallists occurred in stature (0.04 m), last four strides before take-off (0.01–0.09 m), and foot contact time (0.01 s) and projection angle at take-off (0.01°). Compared with non-medallists, notable differences for women occurred with medallists attaining a greater mean velocity from 11–6 m (0.16 m/s) and angle of projection (1.48°) and vertical velocity at take-off (0.23 m/s). Similarities for the women’s medallists and non-medallists occurred in contact time (0 s) and horizontal velocity at take-off (0.03 m/s).

**Table 11:** Summary of long jump competition analysis for men and women, grouped as medallists and non-medallists [16, 31, 59]

Long jump		Men					Women				
Variable		Medallist		Non-medallist			Medallist		Non-medallist		
		*n*	Mean (SD)	*n*	Mean (SD)	Difference^a^	*n*	Mean (SD)	*n*	Mean (SD)	Difference^a^
Jump distance (m)		9	8.43 (0.17)	15	8.15 (0.08)	0.39	6	6.96 (0.11)	11	6.72 (0.08)	0.24
Stature (m)		3	1.85 (0.05)	5	1.81 (0.06)	0.4					
Toe to board distance (m)		3	0.15 (0.04)	5	0.1 (0.12)	0.05	3	0.11 (0.05)	6	0.06 (0.04)	0.06
Length of strides prior to take-off (m)											
4		3	2.29 (0.12)	5	2.29 (0.05)	− 0.01					
3		8	2.37 (0.12)	13	2.36 (0.13)	0.01	6	2.11 (0.14)	11	2.2 (0.2)	− 0.08
2		9	2.5 (0.13)	15	2.42 (0.14)	0.09	6	2.18 (0.25)	10	2.15 (0.19)	0.04
1		9	2.17 (0.17)	15	2.22 (0.12)	− 0.05	6	2.27 (0.13)	11	2.34 (0.21)	− 0.07
Velocity of strides prior to take-off (m)											
4		3	10.47 (0.21)	5	10.02 (0.24)	0.45					
3		3	11.1 (0.35)	5	10.56 (0.18)	0.54					
2		6	10.96 (0.37)	10	10.5 (0.34)	0.45	3	9.55 (0.33)	5	9.45 (0.13)	0.1
1		6	10.68 (0.37)	10	10.36 (0.25)	0.31	3	9.35 (0.23)	5	9.31 (0.19)	0.04
Horizontal velocity at touchdown prior to take-off (m/s)		3	10.92 (0.18)	5	10.51 (0.21)	0.41	3	9.72 (0.06)	6	9.42 (0.29)	0.3
Take-off											
Contact time (s)		3	0.11 (0)	5	0.12 (0)	− 0.01	3	0.12 (0.01)	5	0.12 (0.01)	0
Angle (deg)		9	22.2 (2.9)	15	22.21 (3.61)	− 0.01	6	24.67 (3.14)	11	23.18 (3.43)	1.48
Horizontal velocity (m/s)		9	9.04 (0.32)	15	8.76 (0.37)	0.29	6	7.99 (0.28)	11	7.96 (0.33)	0.03
Vertical velocity (m/s)		9	3.51 (0.23)	15	3.4 (0.3)	0.11	6	3.29 (0.2)	11	3.05 (0.21)	0.23
Mean velocity (m/s)											
11–6 m		3	5	10.66 (0.36)	10.27 (0.08)	0.39	3	9.41 (0.32)	5	9.25 (0.2)	0.16
6–1 m		3	5	10.57 (0.32)	10.28 (0.22)	0.29	3	9.42 (0.34)	5	9.35 (0.17)	0.07

*deg* degree, *m* metres, *m/s* metres per second, *n* = number of athletes, *s* seconds

^a^Difference = medallist—non-medallist

Two field-based papers reported on men only [60, 61]. An elite male athlete was characterised by a combined average of competition and training jumps [60]. Variables included height (1.88 m), body mass (80 kg), take-off velocity (9.56 ± 0.4 m/s) and take-off angle (21.4 ± 1.6°). Bridgett and Linthorne [61] also characterised an elite male long jumper through the reporting of variables such as mean (of 22 jumps) approach velocity (10.4 ± 0.3 m/s) and knee angle at touchdown before take-off (167 ± 4°).

#### Triple Jump

Data for the triple jump were extracted from the competition-based (*n* = 7) and laboratory-based (*n* = 1) literature (Table 2). A comparison between medallists and non-medallists during competition is presented in Table 12. All competition-based papers reported an individual analysis in triple jump for a total of 31 men (14 medallists and 17 non-medallists) and 28 women (11 medallists and 17 non-medallists) [17, 18, 31, 63–66]. The greatest differences for men occurred with medallists attaining higher horizontal velocity in the hop (0.28 m/s) and step (0.30 m/s) and vertical velocity in the jump phase (0.14 m/s) in addition to take-off angle from the jump (2.50°). Non-medallists achieved a greater vertical velocity (0.10 m/s) and take-off angle in the hop phase (1.75°). Similarities for men occurred in the horizontal velocity of jump phase (0.03 m/s) and contact time across the hop (0.01 s), step (0 s) and jump (0 s) phases. Medal-winning women achieved consistently higher horizontal velocities in the final two steps (0.35 and 0.23 m/s) and across hop (0.26 m/s), step (0.32 m/s) and jump (0.25 m/s) phases in addition to higher vertical velocity in hop (0.12 m/s) and jump (0.17 m/s). Similarities for women occurred in vertical velocity of step phase (0.04 m/s) and contact time across the hop (0 s), step (0.01 s) and jump (0.01 s) phases.

**Table 12:** Summary of triple jump competition analysis for men and women, grouped as medallists and non-medallists [17, 18, 31, 63–66]

	Men					Women				
Variable	Medallist		Non-medallist			Medallist		Non-medallist		
	*n*	Mean (SD)	*n*	Mean (SD)	Difference^a^	*n*	mean (SD)	*n*	mean (SD)	Difference^a^
Jump distance (m)	14	17.76 (0.30)	17	17.18 (0.21)	0.58	11	14.81 (0.28)	17	14.28 (0.29)	0.53
Toe to board distance (m)	3	0.12 (0.02)	5	0.23 (0.1)	− 0.11	4	0.09 (0.02)	8	0.10 (0.04)	− 0.01
Stride length before take-off (m)										
− 2	7	2.47 (0.11)	13	2.5 (0.23)	− 0.03	8	2.29 (0.13)	14	2.26 (0.18)	0.03
− 1	7	2.33 (0.09)	13	2.42 (0.18)	− 0.10	9	2.24 (0.23)	17	2.15 (0.19)	0.09
Horizontal velocity (m/s)										
− 2	7	10.21 (0.16)	13	10.09 (0.18)	0.12	8	9.38 (0.31)	14	9.04 (0.20)	0.35
− 1	10	10.38 (0.24)	14	10.28 (0.25)	0.10	9	9.18 (0.39)	17	8.95 (0.40)	0.23
Hop	10	9.69 (0.30)	14	9.41 (0.43)	0.28	9	8.42 (0.23)	17	8.16 (0.33)	0.26
Step	10	8.63 (0.42)	14	8.33 (0.30)	0.30	9	7.65 (0.35)	17	7.32 (0.45)	0.32
Jump	10	7.02 (0.40)	14	6.99 (0.22)	0.03	9	6.45 (0.4)	17	6.2 (0.43)	0.25
Vertical velocity (m/s)										
Hop	10	2.23 (0.31)	14	2.33 (0.22)	− 0.10	9	2.37 (0.19)	17	2.24 (0.29)	0.12
Step	10	1.96 (0.19)	14	1.98 (0.22)	− 0.01	9	1.58 (0.21)	17	1.62 (0.25)	− 0.04
Jump	10	2.71 (0.20)	14	2.57 (0.31)	0.14	9	2.47 (0.19)	17	2.30 (0.25)	0.17
Contact time (s)										
Hop	4	0.13 (0.00)	8	0.12 (0.01)	0.01	6	0.13 (0.02)	12	0.13 (0.02)	0.00
Step	4	0.17 (0.01)	8	0.16 (0.02)	0.00	6	0.15 (0.02)	12	0.16 (0.01)	− 0.01
Jump	4	0.18 (0.01)	8	0.18 (0.02)	0.00	6	0.16 (0.02)	12	0.18 (0.02)	− 0.01
Take-off angle (deg)										
Hop	4	12.50 (2.65)	8	14.25 (1.28)	− 1.75	6	15.42 (1.48)	12	15.43 (1.95)	− 0.02
Step	4	13.25 (0.50)	8	14.13 (1.46)	− 0.88	6	11.92 (1.73)	12	12.88 (1.96)	− 0.97
Jump	4	21.75 (3.30)	8	19.25 (1.49)	2.50	6	20.80 (1.91)	12	20.23 (2.74)	0.57
Distance (m)										
Hop	14	6.10 (0.57)	17	6.3 (0.18)	− 0.20	11	5.40 (0.29)	17	5.29 (0.22)	0.11
Step	14	5.35 (0.19)	17	5.17 (0.22)	0.18	11	4.19 (0.20)	17	4.14 (0.23)	0.05
Jump	14	6.38 (0.65)	17	5.89 (0.27)	0.49	11	5.34 (0.23)	17	4.96 (0.28)	0.38
Relative distance (%)										
Hop	14	34.22 (3.28)	17	36.32 (1.09)	− 2.10	11	36.14 (1.55)	17	36.64 (1.29)	− 0.50
Step	14	30.09 (1.20)	17	29.79 (1.2)	0.30	11	28.15 (1.27)	17	28.78 (1.53)	− 0.62
Jump	14	35.69 (3.41)	17	33.89 (1.49)	1.80	11	35.89 (1.51)	17	34.46 (1.8)	1.43

*Deg* degree, *m* metres, *m/s* metres per second, *n* = number of athletes, *s* = seconds

^a^Difference = medallist—non-medallist

One laboratory-based paper characterised an elite female triple jumper (Olympic finalist) performing 25- and 45-cm drop jumps [22]. The athlete attained jump heights of 49.6 and 51.0 cm for 25- and 45-cm drops jumps, respectively. From a 25-cm drop jump, the athlete achieved a maximal force of 1382 and 1354 N for their left and right leg, respectively, and take-off velocity of 2.88 m/s. The corresponding values from a 45-cm drop were 1482 and 1439 N for the left and right leg and 2.92 m/s at take-off.

## Discussion

The purpose of this study was to systematically review the literature that characterised or differentiated between elite able-bodied speed/power-based track and field athletes in sprint and hurdles events of 400 m and less and the high, long and triple jump. To achieve this, biomechanical, physiological and technical parameters associated with podium success at BME such as the World Championships or Olympic Games were considered. Based on the results of this review alone, the evidence is insufficient to definitively differentiate between medallists and non-medallists; however, characteristics have been identified that, with ongoing investigation, may yield differences in elite performers. Caution should be exercised when interpreting competition analysis within this study. Data collection techniques were not identical across competition analyses because of the variations in locations (i.e. Athens Olympics, Berlin World Championships and regional meets in the USA and Europe), technique (i.e. video capture rate) and technology (i.e. development of laser/radar guns and processing capabilities), so care should be taken when comparing the value of the measure analysed. Attention should be directed to characteristics where differences lie and not necessarily the value of these differences. In addition, each event in this review was considered in isolation with an effort to understand similarities and differences between athletes relative to their event grouping. Key findings emerging from the review are discussed according to the event group.

### 100-m Sprint

In the 100 m, medallists attained a higher peak velocity and average velocity [29, 31, 32] with male medallists generally achieving peak velocity between 60 and 70 m. For medallists in the women’s event, there was no difference in the section of the race where peak velocity was achieved. Notable differences emerged in 10-m split times towards the latter stages of the race [30–32], indicating medallists may accelerate for longer and hold peak velocity for longer, thereby limiting deceleration in the finishing stages of the race. Individual stride parameters were reported for men in one race only [35], so it is difficult to make any inferences based on this; however, grouped results indicate ‘faster’ sprinters may attain a greater stride frequency and length [9]. Additionally, this review found male elite sprinters had greater relative muscle volume, as measured by magnetic resonance imaging, but these results may be confounded by the age of the athlete and so may not be accurate for the profiling of elite sprinters. In addition to papers that allow for an individual analysis at competitions, there are seven papers that focused on one component of the race, or laboratory-based measures, and therefore did not contribute individual athlete data for comparisons between medallists and non-medallists. These papers are useful in augmenting our understanding and profiling the elite athlete, for example, examining the block start set-up of one elite male athlete [29].

Articles that included the 100 m on average were rated as having good methodological quality (69.1 ± 14.0%). It was a strength that the literature to date has examined athletes both within competition- and laboratory-based settings. This allows for a comparison between medal winners and non-medal winners to identify trends relating to potential differences in key areas, and the characterisation of the elite sprinter. Characteristics that may be aligned with BME podium success in the 100 m include peak velocity, the ability to limit deceleration in latter stages of the race and, for men, stride frequency and stride length. Laboratory-based studies often allow for greater control over variables, enabling the isolation and examination of key variables of interest. There is also a greater ability for the standardisation of protocols, which enhances the validity of the research. Additionally, laboratory-based studies eliminate or reduce the impact of environmental factors, contributing to an increase in the reliability of outcomes.

Within the 100-m papers, the strengths of good quality papers generally included clear purpose and relevant background researched. The limitations of these papers often lay in the lack of informed consent and no acknowledgement of limitations; however, given the number of papers that utilised a competition analysis, these limitations are not unexpected.

### 200-m Sprint

In the 200 m, medallists in the women’s race accelerated faster than their non-medal winning counterparts in the first 50 m with notable differences in velocity occurring across the first, second and third 50-m sections [31, 32]. This faster pace held across the initial stages may not transfer into the final 50 m, indicating the first 150 m may be more important than the final 50 m in deciding the outcome of a women’s 200-m race. Men do not share this race strategy and exhibited a different race pattern. The greatest differences in 50-m velocities occurred in the second 50-m and final 50-m sections [31, 32]. This indicates that medal winning athletes in the men’s 200 m take longer to accelerate but are better able to maintain velocity in the closing stages. However, this review found there was insufficient evidence to make further inferences about performance differences between medallists and non-medallists at BME, with only three 200-m specific papers meeting the criteria for inclusion in this review. The three articles examining the 200 m, on average, were scored as having good methodological quality (55.6 ± 7.7%). However, of these studies, two were scored as having good methodological quality and one was scored as poor. These articles examined athletes in a competition-based setting, and while a competition-based analysis is beneficial because athletes are performing at maximum capacity, our understanding of 200-m runners may be enhanced with laboratory-based studies to complement findings. Laboratory-based studies limit the confounding variables and enhance the ability to measure key variables of interest. For example, characteristics that may be assessed within a laboratory-based setting include peak velocity or anaerobic speed reserve.

Papers deemed to be good quality provided a clear purpose of the study, description of the sample and methodology. However, common areas that lowered the quality of the papers included limitations not being acknowledged, a limited review of the background literature [31, 32], no justification of sample size [41] and no description of the validity of measures [31, 41].

### 400-m Sprint

In the 400 m for men, notable areas where differences occurred included the first 100 m, where non-medallists covered the distance faster than medallists [31, 32]. Given these athletes did not go on to win the race, this may suggest a poor pacing strategy or inability to maintain velocity under fatigue. Medallists were also better able to maintain velocity throughout the race, with a smaller drop off between peak velocity and the final 50 m than the non-medallists, potentially indicating increased efficiency or aerobic capacity or a greater ability for lactate tolerance [66]. For medallists in the women’s event, the greatest differences from their non-medal winning counterparts emerged in the final 200-m sections [31, 32]. As with the men, this may suggest a greater ability for lactate tolerance or increased efficiency or aerobic capacity. There were little to no differences across the first 200 m of the race for women, suggesting the ability to maintain velocity in the closing stages may be a factor separating the medallists from the non-medallists. Further insights into parameters such as stride patterns are necessary to gain more understanding of the differences between podium success at BME.

Given only two papers, with an average quality score of 53.4 ± 9.4%, met the criteria to be included in this review, there is insufficient evidence to make further inferences, particularly given the lack of consistency in quality scores between the two papers (one scored as good, and one scored as poor). While both papers provided a clear purpose and an appropriate design for the research question, the paper scored as good also reported the practical applications and validity of outcomes [32].

### 100/110-m Hurdles

Medallists in both the men’s (110-m race distance) [31, 33, 45] and women’s (100-m race distance) [31, 33, 43, 44] event reached the first hurdle (~ 13 m from start line) faster than their non-medal winning counterparts, indicating a greater ability to accelerate. This greater ability to accelerate and reach hurdle one first may also be associated with a better start position and/or better start; however, the literature to date does not provide insight into this. Additionally, medallists in the men’s event achieved a consistently higher hurdle unit velocity while the greatest difference in the women’s event emerged from hurdle six onwards. A further notable difference emerged in the section from hurdle 10 touchdown to the finish line, with medallists for both men and women achieving a greater velocity. This may be reflective of a superior technical ability of medallists, specifically a greater ability to maintain clearance ability and balance under fatigue, thereby minimising a loss in velocity. There did not appear to be any notable differences in hurdle clearance times, suggesting elite-level hurdlers have similar hurdling abilities, with differences to the final race position emerging from the running component of the event.

Additional papers that did not allow for an individual analysis instead focused on profiling the start [50], fourth and fifth hurdle [46, 50], or ninth hurdle [42] for women, or, in the men’s event, the fifth hurdle [47, 48] and first 5 m [49]. Articles that included the 100/110-m hurdles on average were rated as having good methodological quality (65.5 ± 18.6%). It was a strength that articles examining this event group included both competition and training analyses, allowing for both comparisons to identify trends between medallists and non-medallists and the profiling of elite hurdlers. Often studies have profiled one hurdle only and state that the one hurdle selected is reflective of the entire race [47, 48]; however, based on the results of this review, this may not be the case.

Common areas that strengthened the quality of papers included providing a justification for the sample size, appropriate analyses methods and appropriate conclusions given the study methodology. Areas where differences emerged between papers included obtaining informed consent and examining the reliability and validity of outcome measures. Generally, papers in the hurdles event groups did not acknowledge the limitations of the study.

### 400-m Hurdles

In the 400-m hurdles, from hurdle four onwards for men and hurdle three onwards for women, medallists attained a lower step count between hurdles [33, 51]. This may reflect a greater ability to maintain velocity and stride length under fatigue to cover the distance in fewer steps than their non-medalling counterparts. Additionally, from hurdle six onwards for men and hurdle five onwards for women, medallists achieved a faster hurdle unit time, suggesting medallists may have a better rhythm or be more efficient and have a greater aerobic capacity, which allows them to better maintain velocity under fatigue. The greatest difference in velocity between medallists and non-medallists for both men and women occurred between hurdle 10 touchdown and the finish line, again supporting the notion that medallists are better able to maintain velocity under fatigue. However, this may also be because of non-medallists ‘pulling up’ before the line as a result of knowing they have no chance of medalling. In the 2000 Olympic 400-m men’s hurdle final, athletes positioned from fifth to eighth at hurdle eight remained in the bottom four at the finish [51]. When step frequency and step length were examined in world class 400-m hurdlers (47.71 ± 0.44 s) and national hurdlers (49.28 ± 0.41 s), the greatest difference between groups emerged in step frequency in the first 200 m (world class: 3.75 ± 0.12 Hz, national: 3.6 ± 0.11 Hz) [52]. Articles examining the 400-m hurdles were scored as good methodological quality (64.4 ± 13.9%). Only three papers [33, 51, 52] examining the 400-m hurdles met the inclusion criteria for this review, all three were scored as having good methodological quality. Areas where differences emerged between papers included the level of detail when reviewing the background literature, the description of the methodology and acknowledgement of the limitations of the study. Characteristics that may be aligned with elite BME performance were identified (i.e. velocity maintenance or step count); however, there was insufficient evidence to differentiate characteristics between medal winning and non-medal performance.

### High Jump

In the high jump, there was a notable disparity in reporting between men and women. Most high jump-related literature analysed men (*n* = 8) compared with women (*n* = 2). Medallists in both the men’s and women’s events achieved a greater vertical take-off velocity than their non-medal winning counterparts [19, 31, 53–56]. Medallists in the women’s event also achieved a greater horizontal velocity at touchdown prior to take-off [53]. When examined in relation to the greater vertical velocity at take-off, this suggests a greater ability to transfer horizontal velocity to vertical. However, because of the lack of data on the mean or peak approach velocity, it is difficult to infer more that could lead to a clearer understanding of characteristics that may differentiate between medallists and non-medallists. Medal winning men and non-medal winning women achieved a greater peak arm velocity and arm velocity at take-off than their counterparts [53]. Further work may be required to better understand the impact of this and how this may differ between men and women. Medal winning men achieved a greater vertical velocity at touchdown prior to take-off and at take-off [54, 55]. This indicates men may begin the transfer of horizontal velocity to vertical earlier in preparation for take-off; however, further information on approach velocity is required to confirm this.

While two papers did not contribute to individual competition-based analyses, they potentially allow for the profiling of male athletes [57, 58]. These papers focused on approach velocity in addition to the final contact, specifically the arm action, vertical velocity at take-off, plant angle and knee angle at touchdown.

Overall, articles examining the high jump achieved an average quality score of 62.5 ± 14.7%, indicating good methodological quality. All papers clearly stated the purpose of the study and described the participants in detail. However, areas where differences between poor and excellent quality emerged included the detail in the description of the methodology, the reporting of results in terms of statistical significance and the acknowledgement of limitations. These articles consisted of a competition-based analysis only, thereby identifying multiple characteristics associated with BME performance, such as vertical take-off velocity or horizontal velocity at touchdown before take-off. However, the relationships of these characteristics to medal winning performances at BME are still unclear.

### Long Jump

In the long jump event, there were several key areas where differences emerged between medallists and non-medallists. Medallists for both women and men attained a greater mean approach velocity in both the 11–6 m and 6–1 m sections [59]. Medallists in the men’s event achieved a greater velocity in each stride across the last four strides [16, 59]. This suggests a greater ability to accelerate, maximum velocity capacity and a technical ability to control velocity on the runway. More control on the runway enables the athlete to spot the board on the approach and limit adjustments in the final strides that would result in velocity loss. Both men and women medallists achieved a greater horizontal velocity at touchdown prior to take-off [60]. However, this variable was only reported in one paper; therefore, it is difficult to infer more because of the low sample size. At take-off, vertical velocity was greater for medallists in both the men’s and women’s events [16, 31, 59], while horizontal velocity was only greater for men [16, 31]. This may require further investigation, particularly for women, given the goal of the event is to cover the greatest horizontal distance. Two further papers examining approach velocity, take-off velocity, take-off angle, knee angle at take-off touchdown, take-off height and take-off distance [60, 61] may allow for further athlete profiling but did not enable comparisons between medallists and non-medallists.

Articles examining long jump achieved an average quality score of 58.7 ± 8.7%, classed as having good methodological quality. Areas that contributed to papers achieving a good quality score included implementing a research design that was appropriate for the research question and reporting the practical importance of the paper. Inconsistency in the reporting of the reliability and validity of outcome measures and in the justification of sample sizes, in addition to no acknowledgement of limitations across any of the included papers, were factors that impacted the quality score. It was a strength that articles in this event group examined athletes both in and out of competition. This allowed for a comparison between medal winning and non-medal winning athletes to identify trends in characteristics, such as the ability to accelerate or maximise velocity, which are potentially associated with better BME performance. Additionally, the measurement of characteristics not commonly assessed in competition (i.e. take-off angle) allows for further characterising of elite long jumpers that may be key factors in separating medallists from non-medallists.

### Triple Jump

In the triple jump, a smaller toe-to-board distance was noted with medallists in the men’s event only [31]. This may indicate a greater ability to sight the board and make any necessary stride adjustments during the approach phase. Medallists in the women’s event attained a greater horizontal velocity from two steps prior to take-off through to the jump phase take-off [17, 31, 65], thereby demonstrating a greater ability to maintain velocity across the phases of the triple jump than their non-medal winning counterparts. In the men’s event, medallists recorded a greater horizontal velocity from two steps prior to take-off through to the step phase take-off, with no notable difference observed in horizontal take-off velocity at the jump phase [18, 31, 62]. However, both men and women medallists achieved a greater vertical velocity at take-off for the jump phase than the non-medallists [17, 18, 31, 55, 58]. Finally, it appears as though medallists in both the men’s and women’s events are jump dominant, evident in the greatest relative distance of their overall triple jump distance occurring in the jump phase [11, 12, 24, 62–65]. In contrast, non-medallists were found to be hop dominant, attaining a greater relative distance in the hop phase. In addition to the competition-based analysis, an elite female triple jumper was profiled across both 25- and 45-cm drop jumps, with parameters such as force, impulse, jump height and contact time reported [22]. While this does not allow for a direct comparison between medallists and non-medallists, it does allow for the profiling of an elite woman in a common jump test.

With an average methodological quality score of 62.5 ± 10.6%, articles examining triple jump were found to be of good methodological quality. Papers that scored as good or excellent quality generally stated a clear purpose, reviewed the relevant background literature and made appropriate conclusions given the methodology. Areas that separated excellent from good included describing the reliability and validity of outcome measures in addition to acknowledging limitations of the study. Therefore, when examining characteristics identified through a competition-based analysis, it is important to acknowledge that while potential areas that differentiate between BME success may emerge, these differences are not definitive at this stage.

### Findings Summary

Across the speed/power-based sprint and jump disciplines, medal winning athletes consistently demonstrate superior acceleration, greater peak velocities and greater efficiency in maintaining velocity under fatigue. In the sprint events, medal winning athletes optimise step mechanics, and sustain velocity more effectively, particularly in latter race phases, relative to their non-medal winning counterparts. Medal winning long and triple jumpers achieve greater approach velocities while demonstrating technical precision at take-off, balancing velocity with optimal positioning to maximise jump distance. Medal-winning high jumpers consistently demonstrate superior take-off mechanics, achieving a more optimal combination of approach velocity, force application and body positioning at the point of take-off to maximise the centre of mass height above bar and bar clearance. Additionally, the hurdle-based events medal winners were better able to maintain velocity maintenance between hurdles, suggesting technical execution and fatigue resistance are important. While physiological traits such as neuromuscular efficiency, reactive strength and tendon stiffness likely contribute to podium success in speed/power-based track and field athletes, further research is needed to clarify their direct impact. Future training should emphasise individualised approaches to optimising speed, technique and fatigue management, while research should work toward standardised methodologies and a deeper understanding of psychological and social influences on elite performance.

Additionally, while this review includes speculation and caveats, this reflects the reality of the existing literature. The lack of definitive evidence on many of these characteristics is itself a critical finding, highlighting the need for further investigation. Within applied sport and high-performance settings, decisions about athlete development, talent identification and training are often made based on an incomplete and fragmented evidence base. By structuring and synthesising the available research, this review addresses this gap and helps guide more informed decision making. For example, while large datasets, such as those from World Athletics, offer convenience, they do not necessarily clarify which variables truly differentiate performance. Rather than assume commonly reported variables are the most relevant, this review took a step back to assess the strength of the existing evidence. This approach ensures that future research and applied practice are built on a more rigorous foundation.

### Limitations

This systematic review has some limitations that should be considered when interpreting the results. A key challenge in this area is that much of the existing research relies on data collected from competition reporting, which inherently limits methodological quality. As a result, several studies within this review were characterised as good-to-low quality. These studies lack the experimental control or longitudinal depth needed for stronger causal inferences. However, this is an unavoidable consequence of researching elite performance, where experimental interventions are difficult to implement, and many of the available data are derived from observational studies and retrospective analyses. Rather than dismissing this body of work, the present review sought to critically assess and consolidate what is currently available. Additionally, the peak velocity reported is an average for a 10-m section and not peak velocity at a single timepoint [29, 31–33, 35]. This therefore may not be an accurate reflection of an athlete’s true peak velocity. Some race analysis characteristics may be deemed convenience measures (i.e. easy to measure within the constraints of the environment) and not necessarily assessed because they are linked to performance outcomes. Papers may have noted a difference between groups within their study; however, the study may have compared world-class athletes to academy, national or lower-level athletes [22, 23]. Therefore, observed differences are not unexpected. The competition data in this review were collected using a two-dimensional video across a range of capture rates. These include 25 Hz [51], 50 Hz [19, 31–33, 53, 54], 60 Hz [30, 55, 63, 64], 100 Hz [16, 32, 41, 44, 45, 62, 65], 200 Hz [29, 54], 250 Hz [55] and 300 Hz [17, 18, 34, 35, 56, 59]. Finally, the limited sample sizes for some event groups (i.e. 200 m, 400 m, 400-m hurdles and women’s high jump) may have impacted the generalisability of results for these event groups and we were unable to compute standardised effects because of the small group numbers.

## Conclusions

This systematic review aimed to assess current measures that may be used to identify and/or differentiate parameters associated with podium success in able-bodied track and field athletes in speed/power-based events. Various characteristics, across the eight event groups examined in this review, that may be associated with BME medal-winning performances were identified. Examples of characteristics identified included, but were not limited to, peak velocity, ability to accelerate for longer and to maintain velocity, step frequency, hurdle step count, runway approach velocity, horizontal take-off velocity and vertical take-off velocity. It remains uncertain whether these characteristics can effectively differentiate between medallists and non-medallists. This outcome, however, can be used to guide performance testing, as characteristics associated with BME podium success have been identified. Furthermore, by providing an indication of the thresholds they are trying to achieve, this review can be used to inform performance gaps or areas for development in emerging athletes. These data cannot be used to definitively identify which characteristics will differentiate or predict an athlete’s ability to achieve podium success at BME. Additionally, it is important to note the presence of bias in the literature towards reporting on the shorter sprint events and men’s high jump. These findings underscore the need for more comprehensive and unbiased research, grounded in a strong theoretical framework, in order to gain a deeper understanding of the factors influencing BME success.

## Declarations

### Availability of Data and Material

Data relevant to the study are included in the article or available on request from the corresponding author.

## References

[1] Simonton DK. Talent and its development: an emergenic and epigenetic model. Psychol Rev. 1999;106(3):435–57. 10.1037/0033-295X.106.3.435.

[2] Pickering C, Kiely J. Can the ability to adapt to exercise be considered a talent—and if so, can we test for it? Sports Med Open. 2017;3(1):43. 10.1186/s40798-017-0110-3. 29188457 10.1186/s40798-017-0110-3 PMC5707216

[3] Baker J, Wattie N, Schorer J. A proposed conceptualization of talent in sport: the first step in a long and winding road. Psychol Sport Exerc. 2019;43:27–33. 10.1016/j.psychsport.2018.12.016.

[4] Ericsson KA. Deliberate practice and the modifiability of body and mind: toward a science of the structure and acquisition of expert and elite performance. Int J Sport Psychol. 2007;38(1):4–34.

[5] Hollings SC, Clifford MJ, Hume PA. The transition for elite junior track-and-field athlete to successful senior athlete: why some do, why others don’t. Int J Sports Sci Coach. 2014;9(3):457–71. 10.1260/1747-9541.9.3.457.

[6] Boccia G, Cardinale M, Brustio PR. World-class sprinters’ careers: early success does not guarantee success at adult age. Int J Sports Physiol Perform. 2021;16(3):367–74. 10.1123/ijspp.2020-0090. 33296871 10.1123/ijspp.2020-0090

[7] Leite N, Calvo AL, Cumming S, Gonçalves B, Calleja-Gonzalez J. Talent identification and development in sports performance. Front Sports Act Living. 2021;3:1–4. 10.3389/fspor.2021.729167. 10.3389/fspor.2021.729167 PMC8652003 34901848

[8] Majumdar A, Robergs R. The science of speed: determinants of performance in the 100 m sprint. Int J Sports Sci Coach. 2011;6(3):479–93. 10.1260/1747-9541.6.3.479.

[9] Paruzel-dyja M, Walaszczyk A, Iskra J. Elite male and female sprinters’ body build, stride length and stride frequency. Stud Phys Cult Tour. 2006;13(1):33–7.

[10] Chelly SM, Denis C. Leg power and hopping stiffness: relationship with sprint running performance. Med Sci Sports Exerc. 2001;33(2):326–33. 10.1097/00005768-200102000-00024. 11224825 10.1097/00005768-200102000-00024

[11] McAuley ABT, Baker J, Kelly AL. Defining “elite” status in sport: from chaos to clarity. Ger J Exerc. 2022;52(1):193–7. 10.1007/s12662-021-00737-3. 10.1007/s12662-021-00737-3 PMC8340584 40477382

[12] Swann C, Moran A, Piggott D. Defining elite athletes: issues in the study of expert performance in sport psychology. Psychol Sport Exerc. 2015;16:3–14. 10.1016/j.psychsport.2014.07.004.

[13] McKay AKA, Stellingwerff T, Smith ES, Martin DT, Mujika I, Goosey-Tolfrey VL, et al. Defining training and performance caliber: a participant classification framework. Int J Sports Physiol Perform. 2022;17(2):317–31. 10.1123/ijspp.2021-0451. 34965513 10.1123/ijspp.2021-0451

[14] Amara S, Mkaouer B, Chaabene H, Negra Y, Ben-Salah FZ. Key kinetic and kinematic factors of 110-m hurdles performance. J Phys Educ Sport. 2019;19(1):658–68. 10.7752/jpes.2019.01095.

[15] Salo A, Grimshaw PN, Marar L. 3-D biomechanical analysis of sprint hurdles at different competitive levels. Med Sci Sports Exerc. 1997;29(2):231–7. 9044228 10.1097/00005768-199702000-00011

[16] Hay JG, Miller JA, Canterna RW. The techniques of elite male long jumpers. J Biomech. 1986;19(10):855–66. 10.1016/0021-9290(86)90136-3. 3782168 10.1016/0021-9290(86)90136-3

[17] Pavlović R, de Oliveira WM. The differences of kinematic parameters triple jump between female finalist’s World Championship Berlin, 2009-Daegu, 2011. Turk J Kinesiol. 2017;3(4):60–9.

[18] Pavlović R. The differences of kinematic parameters triple jump between finalists WCH Berlin, 2009-WCH Daegu, 2011. Eur J Phys Educ Sport. 2018;6(1):20–30. 10.13187/ejpe.2018.1.20.

[19] Lees A, Rojas J, Cepero M, Soto V, Gutierrez M. How the free limbs are used by elite high jumpers in generating vertical velocity. Ergonomics. 2000;43(10):1622–36. 10.1080/001401300750004041. 11083142 10.1080/001401300750004041

[20] Panoutsakopoulos V, Kollias IA. 3D Biomechanical analysis of women’s high jump technique. New Studies Athletics. 2012;27(3):31–44.

[21] Pavlović R. The morphological status of the finalist in jumping disciplines at the Beijing Olympics. Sport Sci. 2012;5(2):43–8. 10.13140/RG.2.1.2210.7681.

[22] Čoh M, Matjačić Z, Peharec S, Bačić P, Rausavjević N, Maćkala K. Kinematic, dynamic and EMG analysis of drop jumps in female elite triple jump athletes. Coll Antropol. 2015;39(1):159–66. 26434025

[23] Miller R, Balshaw TG, Massey GJ, Maeo S, Lanza MB, Johnston M, et al. The muscle morphology of elite sprint running. Med Sci Sports Exerc. 2021;53(4):804–15. 10.1249/MSS.0000000000002522. 33009196 10.1249/MSS.0000000000002522

[24] Moher D, Liberati A, Tetzlaff J, Altman DG. Preferred reporting items for systematic reviews and meta-analyses: the PRISMA statement. BMJ. 2009;339(7716):332–6. 10.1136/bmj.b2535. 10.1136/bmj.b2535 PMC2714657 19622551

[25] Sarmento H, Clemente FM, Araújo D, Davids K, McRobert A, Figueiredo A. What performance analysts need to know about research trends in Association Football (2012–2016): a systematic review. Sports Med. 2018;48:799–836. 29243038 10.1007/s40279-017-0836-6

[26] Low B, Coutinho D, Gonçalves B, Rein R, Memmert D, Sampaio J. A systematic review of collective tactical behaviours in football using positional data. Sports Med. 2020;50(2):343–85. 10.1007/s40279-019-01194-7. 31571155 10.1007/s40279-019-01194-7

[27] Messias LHD, Reis IGMD, Bielik V, Garbuio ALP, Gobatto CA, Manchado-Gobatto FB. Association between mechanical, physiological, and technical parameters with canoe slalom performance: a systematic review. Front Physiol. 2021;12:1–10. 10.3389/fphys.2021.734806. 10.3389/fphys.2021.734806 PMC8637211 34867443

[28] Law M, Stewart D, Pollock N, Letts L, Bosch J, Westmorland M. Critical review form: quantitative studies. Hamilton: McMaster University; 1998.

[29] Moravec P, Ruzicka J, Susanka P, Dostal E, Kodejs M, Nosek M. The 1987 International Athletic Foundation/IAAF Scientific Project Report: time analysis of the 100 metres events at the II World Championships in Athletics. New Studies Athletics. 1988;3:61–96.

[30] Ae M, Ito M, Suzuki M. The men’s 100 metres. New Studies Athletics. 1992;7(1):47–52.

[31] Müller H, Honinicl I. Biomechanical research project at the VIth World Championships in Athletics, Athens 1997: preliminary report. New Studies Athletics. 1997;12(2–3):43–73.

[32] Ferro A, Rivera A, Pagola I, Ferreruela M, Martin Ä, Rocandio V. Biomechanical analysis of the 7th world championships in athletics Seville 1999. New Studies Athletics. 2001;16(1/2):25–60.

[33] Graubner R, Nixdorf E. Biomechanical analysis of the sprint and hurdles events at the 2009 IAAF world championships in athletics. New Studies Athletics. 2011;26:19–53.

[34] Taylor MJD, Beneke R. Spring mass characteristics of the fastest men on Earth. Int J Sports Med. 2014;33:667–70. 10.1055/s-0032-1324404. 10.1055/s-0032-1306283 22510798

[35] Čoh M, Hébert-Losier K, Štuhec S, Babić V, Supej M. Kinematics of Usain Bolt’s maximal sprint velocity. Kinesiology. 2018;50(2):172–80. 10.26582/K.50.2.10.

[36] Salo AIT, Bezodis IN, Batterham AM, Kerwin DG. Elite sprinting: are athletes individually step-frequency or step-length reliant? Med Sci Sports Exerc. 2011;43(6):1055–62. 10.1249/MSS.0b013e318201f6f8. 20980924 10.1249/MSS.0b013e318201f6f8

[37] Krzysztof M, Mero A. A kinematics analysis of three best 100 M performances Ever. J Hum Kinet. 2013;37:149–61. 10.2478/hukin-2013-0015 PMC3661886 23717364

[38] Ciacci S, Merni F, Bartolomei S, di Michele R. Sprint start kinematics during competition in elite and world-class male and female sprinters. J Sports Sci. 2017;35(13):1270–8. 10.1080/02640414.2016.1221519. 27540875 10.1080/02640414.2016.1221519

[39] Slawinski J, Termoz N, Rabita G, Guilhem G, Dorel S, Morin JB, et al. How 100-m event analyses improve our understanding of world-class men’s and women’s sprint performance. Scand J Med Sci Sports. 2017;27(1):45–54. 26644061 10.1111/sms.12627

[40] Colyer SL, Graham-Smith P, Salo AIT. Associations between ground reaction force waveforms and sprint start performance. Int J Sports Sci Coach. 2019;14(5):658–66.

[41] Mann R, Herman J. Kinematic analysis of Olympic sprint performance: men’s 200 meters. Int J Sport Biomech. 1985;1(2):151–62.

[42] Mann R, Herman J. Kinematic analysis of Olympic hurdle performance: women’s 100 meters. J Appl Biomech. 1985;1(2):163–73. 10.1123/ijsb.1.2.163.

[43] Hucklekemkes J. Model technique analysis sheets for the hurdles PART VI: the Women’s 100 metres hurdles. New Stud Athl. 1990;5(4):33–58.

[44] Tsiokanos A, Tsaopoulos D, Giavroglou A, Tsarouchas E. Race pattern of women’s 100-m hurdles: time analysis of Olympic hurdle performance. Int J Kinesiol Sports Sci. 2017;5(3):56. 10.7575/aiac.ijkss.v.5n.3p.56.

[45] Tsiokanos A, Tsaopoulos D, Tsarouchas E, Giavroglou A. Race pattern of men’s 110-M hurdles: time analysis of Olympic hurdle performance. Biol Exerc. 2018;14(2):15–36. 10.4127/jbe.2018.0136.

[46] Čoh M, Dolonec A. Three-dimensional kinematic analysis of the hurdles technique used by Brigita Bukovec. New Studies Athletics. 1996;11(1):63–9.

[47] Amara S, Mkaouer B, Chaabène H, Negra Y, Hammoudi-Riahi S, Ben-Salah FZ. Kinetic and kinematic analysis of hurdle clearance of an African and a world champion athlete: a comparative study. South Afr J Res Sport Phys Educ Recreat. 2017;39(2):1–12.

[48] Čoh M, Bončina N, Štuhec S, Mackala K. Comparative biomechanical analysis of the hurdle clearance technique of Colin Jackson and Dayron Robles: key studies. Appl Sci. 2020;10(9):3302. 10.3390/app10093302.

[49] Charalambous L, Irwin G, Bezodis IN, Kerwin D. Lower limb joint kinetics and ankle joint stiffness in the sprint start push-off. J Sports Sci. 2012;30(1):1–9. 22098532 10.1080/02640414.2011.616948

[50] Rash GS, Garrett J, Voisin M. Kinematic analysis of top American female 100-meter hurdlers. Int J Sport Biomech. 1990;6:386–94.

[51] Ditroilo M, Marini M. Analysis of the race distribution for male 400m hurdlers competing at the 2000 Sydney Olympic Games. New Studies Athletics. 2001;16(3):15–30.

[52] Otsuka M, Isaka T. Intra-athlete and inter-group comparisons: running pace and step characteristics of elite athletes in the 400-m hurdles. PLoS ONE. 2019;14(3):1–17. 10.1371/journal.pone.0204185. 10.1371/journal.pone.0204185 PMC6438499 30921329

[53] Brüggemann GP, Loch M. The high jump. New Studies Athletics. 1992;7(1):67–72.

[54] Isolehto J, Virmavirta M, Kyröläinen H, Komi P. Biomechanical analysis of the high jump at the 2005 IAAF World Championships in Athletics. New Studies Athletics. 2007;22(2):17–27.

[55] Ae M, Nagahara R, Ohshima Y, Koyama H, Takamoto M, Shibayama K. Biomechanical analysis of the top three male high jumpers at the 2007 World Championships in Athletics. New Studies Athletics. 2008;23(2):45–52.

[56] Pavlović R. The differences of kinematic parameters high jump between male and female finalists World Championship Daegu, 2011. Turk J Kinesiol. 2017;3(4):60–9.

[57] Dapena J, McDonald C, Cappaert J. A regression analysis of high jumping technique. Int J Sport Biomech. 1990;6:246–61. 10.1123/ijsb.6.3.246.

[58] Greig MP, Yeadon MR. The influence of touchdown parameters on the performance of a high jumper. J Appl Biomech. 2000;16(4):367–78. 10.1123/jab.16.4.367.

[59] Pavlović R, Bonacin D, Stankovic D. Differences in kinematic parameters of the long jump between male and female finalists of World Championships-Berlin 2009. Int J Sci Culture Sport. 2016;4(21):353. 10.14486/intjscs528.

[60] Linthorne NP, Guzman MS, Bridgett LA. Optimum take-off angle in the long jump. J Sports Sci. 2005;23(7):703–12. 10.1080/02640410400022011. 16195020 10.1080/02640410400022011

[61] Bridgett LA, Linthorne NP. Changes in long jump take-off technique with increasing run-up speed. J Sports Sci. 2006;24(8):889–97. 10.1080/02640410500298040. 16815784 10.1080/02640410500298040

[62] Miller JA, Hay JG. Kinematics of a world record and other world-class performances in the triple jump. Int J Sport Biomech. 1986;2(4):272–88. 10.1123/ijsb.2.4.272.

[63] Yu B, Hay JG. Optimum phase ratio in the triple jump. J Biomech. 1996;29(10):1283–9. 10.1016/0021-9290(96)00048-6. 8884473 10.1016/0021-9290(96)00048-6

[64] Yu B. Horizontal-to-vertical velocity conversion in the triple jump. J Sports Sci. 1999;17(3):221–9. 10.1080/026404199366. 10362389 10.1080/026404199366127

[65] Panoutsakopoulos V, Kollias IA. Essential parameters in female triple jump technique. New Studies Athletics. 2008;23(4):53–61.

[66] Joyner MJ, Coyle EF. Endurance exercise performance: the physiology of champions. J Physiol. 2008;586(1):35–44. 10.1113/jphysiol.2007.143834. 17901124 10.1113/jphysiol.2007.143834 PMC2375555

